# Ubiquitin ligase EL5 maintains the viability of root meristems by influencing cytokinin-mediated nitrogen effects in rice

**DOI:** 10.1093/jxb/eru110

**Published:** 2014-03-24

**Authors:** Susumu Mochizuki, Yusuke Jikumaru, Hidemitsu Nakamura, Hanae Koiwai, Keisuke Sasaki, Yuji Kamiya, Hiroaki Ichikawa, Eiichi Minami, Yoko Nishizawa

**Affiliations:** ^1^Genetically Modified Organism Research Center, National Institute of Agrobiological Sciences, Kannondai 2-1-2, Tsukuba, Ibaraki, 305-8602Japan; ^2^Growth Regulation Research Group, RIKEN Plant Science Center, Suehiro-cho 1-7-22, Tsurumi, Yokohama, Kanagawa, 230-0045Japan; ^3^Division of Plant Sciences, National Institute of Agrobiological Sciences, Kannondai 2-1-2, Tsukuba, Ibaraki, 305-8602Japan; ^4^National Institute of Livestock and Grassland Science, Ikenodai 2, Tsukuba, Ibaraki, 305-0901Japan

**Keywords:** Cytokinin, nitrogen, reactive oxygen species, rice, root cell death, ubiquitin ligase.

## Abstract

Root formation is dependent on meristematic activity and is influenced by nitrogen supply. We have previously shown that ubiquitin ligase, EL5, in rice (*Oryza sativa*) is involved in the maintenance of root meristematic viability. When mutant EL5 protein is overexpressed to dominantly inhibit the endogenous EL5 function in rice, primordial and meristematic necrosis ia observed. Here, we analysed the cause of root cell death in transgenic rice plants (mEL5) overexpressing *EL5V162A*, which encodes a partly inactive ubiquitin ligase. The mEL5 mutants showed increased sensitivity to nitrogen that was reflected in the inhibition of root formation. Treatment of mEL5 with nitrate or nitrite caused meristematic cell death accompanied by browning. Transcriptome profiling of whole roots exhibited overlaps between nitrite-responsive genes in non-transgenic (NT) rice plants and genes with altered basal expression levels in mEL5. Phytohormone profiling of whole roots revealed that nitrite treatment increased cytokinin levels, but mEL5 constitutively contained more cytokinin than NT plants and showed increased sensitivity to exogenous cytokinin. More superoxide was detected in mEL5 roots after treatment with nitrite or cytokinin, and treatment with an inhibitor of superoxide production prevented mEL5 roots from both nitrite- and cytokinin-induced meristematic cell death. These results indicate a nitrogen-triggered pathway that leads to changes in root formation through the production of cytokinin and superoxide, on which EL5 acts to prevent meristematic cell death.

## Short statement

A rice ubiquitin ligase plays a role in preventing root meristematic cell death in the nitrogen-triggered pathway that leads to the production of cytokinin and superoxide.

## Introduction

Root formation affects vigour and the final yield of crop plants. Nitrogen status influences root growth and development (Forde, 2002; Osmont *et al.*, 2007). Inorganic nitrogen is available in the soil as ammonium, nitrite, and nitrate. Nitrate absorbed by roots is reduced to nitrite by nitrate reductase, and then to ammonium by nitrite reductase in roots and shoots. In general, root elongation and lateral root development are stimulated at low-nitrogen concentrations, while high-nitrogen concentrations confer inhibitory effects on root growth. Several phytohormones are involved in the alteration of root growth in response to nitrogen sources (Aloni *et al.*, 2006; Osmont *et al.*, 2007). *Arabidopsis* (*Arabidopsis thaliana*) NRT1.1 is the nitrate transporter that also facilitates uptake of auxin, and this transporter is proposed to connect nitrate availability and auxin signalling during changes in root growth (Krouk *et al.*, 2010). Supply of nitrogen leads to increased cytokinin content in roots; exogenous application of cytokinin downregulates nitrogen-uptake-related genes (Sakakibara *et al.*, 2006). Inhibition of root growth in maize (*Zea mays*) by high nitrate supply is partly attributed to decreased auxin concentrations (Tian *et al.*, 2008) and increased levels of cytokinin in roots (Tian *et al.*, 2005).

Root formation relies on the activity of the root apical meristem (RAM). Cytokinin inhibits root elongation by negatively regulating RAM activity; cytokinin-deficient plants form larger RAMs and more rapidly growing roots (Werner *et al.*, 2003). Ogawa *et al.* (2011) reported that a rice mutant, *rss1* (*rice salt sensitive 1*), exhibited irreversible growth inhibition under saline conditions; RSS1 contributes to the maintenance of meristematic activity and viability in shoots and roots by regulating the G_1_–S transition in the cell cycle. However, the ways in which activity or viability of the RAM is maintained under various changes in environmental conditions are largely unknown.

The Arabidopsis Tóxicos en Levadura (ATL) family is a large, plant-specific family of RING-H2-type ubiquitin ligases (E3). *ATL* genes were found in 24 plant species (including mosses and lycopods), with 91 genes identified in *Arabidopsis* and 121 in rice (Aguilar-Hernández *et al.*, 2011). CNI1/ATL31 in *Arabidopsis* is the only ATL family member whose substrate has been identified. CNI1/ATL31 functions in the carbon/nitrogen response for the seedling growth-phase transition (Sato *et al.*, 2009) and ubiquitinates 14-3-3 protein, one function of which is to regulate nitrate reductase activity (Sato *et al.*, 2011).

Rice *EL5* is a member of the ATL family, with six copies in the genome (RAP-ID: Os02g0559800–Os02g0561800). The RING-H2-finger domain (or RFD) in EL5 interacts with a rice ubiquitin-conjugating enzyme, UBC5b, and thus is essential for E3 activity (Takai *et al.*, 2002). Mutated RING-H2-finger domains show varying decreases in E3 activity depending on the degree of interaction with UBC5b. Replacement of Trp-165 with Ala results in the complete loss of its E3 activity, whereas replacement of Val-162 with Ala causes a reduction in E3 activity *in vitro* (Katoh *et al.*, 2005). Overexpression of *EL5* in transgenic rice plants and attempts to suppress *EL5* by RNAi constructs lead to no evident morphological changes. In contrast, overexpression of mutated *EL5* to prevent the endogenous EL5 function inhibits root formation in an E3-activity-dependent manner: rice transformants constitutively expressing *EL5W165A* exhibit a rootless phenotype when cultured in Murashige and Skoog (MS) medium, whereas plants expressing *EL5V162A* occasionally develop short crown roots with necrotic lateral roots (Koiwai *et al.*, 2007). The phenotype of these transgenic plants strongly suggests that EL5 preferentially acts on root development by maintaining proliferation and viability of the primordial cells in crown and lateral roots. Moreover, the upregulation of *EL5* expression by cytokinin and the similarity between the phenotype of transgenic plants overexpressing the mutated *EL5* and that of cytokinin-treated roots in non-transgenic (NT) rice plants imply that EL5 might be involved in the cytokinin action in roots (Koiwai *et al.*, 2007). These previous results prompted us to analyse the cause of root cell death in transgenic rice plants expressing mutated *EL5*, to elucidate the mechanisms underlying the maintenance of RAM viability during root formation.

Phytohormones influence various cellular processes, including maintenance of the RAM, through production of reactive oxygen species (ROS) and NO (Joo *et al.*, 2001; Pagnussat *et al.*, 2002; Foreman *et al.*, 2003; Jiang *et al.*, 2003; Correa-Aragunde *et al.*, 2004). However, both ROS and NO are implicated in triggering oxidative cell death (Jabs *et al.*, 1996; Delledonne *et al.*, 1998). Because severe necrosis was observed to be centred on the quiescent centre of crown roots in *EL5W165A*-expressing plants (Koiwai *et al.*, 2007), we hypothesized that phytohormone contents and/or regulation of the generation of ROS or NO are altered in the roots of these plants, which results in meristematic cell death. In the present study, we first found that the presence of nitrogen sources in the culture medium was a causal agent of the rootless phenotype. Thus, we analysed nitrogen-induced root cell death by focusing on the global transcriptome profile, hormone contents, and accumulation of ROS and NO. Through comparative analyses between the mutated *EL5*-overexpressing roots and NT roots, we propose a mechanism that prevents the inhibitory effects of nitrogen on root formation in rice.

## Materials and methods

### Plant materials

Rice seeds (*Oryza sativa* L. *japonica* ‘Nipponbare BL no. 2’) were provided by Dr Hiroyuki Satoh of the National Institute of Crop Science, Tsukuba, Japan. Transgenic rice plants of the same cultivar expressing *EL5V162A* (mEL5) were produced as described in Koiwai *et al.* (2007). Because it was difficult to grow mEL5 transformants to the bearing stage due to their deficient roots and because mEL5 shoots were actively branching, we used regenerated mEL5 shoots that were subcultured for a 3 week interval in MS medium containing 1% (w/v) agar, 25 mg·l^−1^ hygromycin, and 6.25 mg·l^−1^ meropen (Dainippon Sumitomo Pharma, Tokyo, Japan) at 28 °C under continuous light (35 µmol·m^−2^·s^−1^) in a growth chamber.

### Root-formation test

**Fig. 1.**
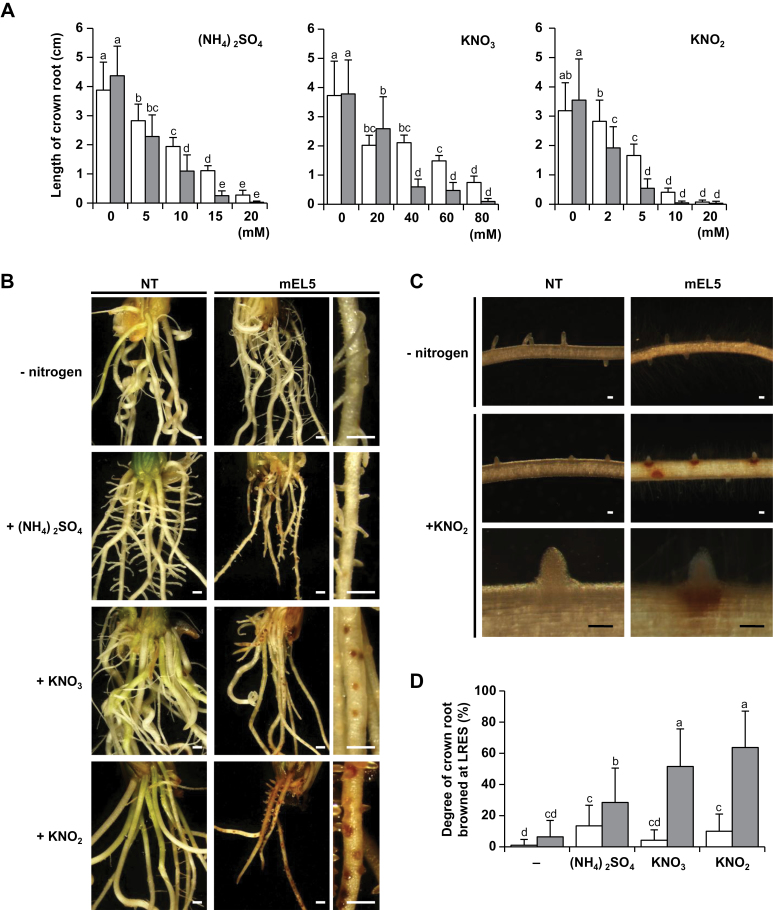
Phenotypes of mEL5 roots in response to nitrogen supply. (A) Inhibitory effect of nitrogen on crown root elongation. Average length of the longest crown root of non-transformants (NT, white bars) and transgenic rice plants overexpressing *EL5V162A* (mEL5, grey bars) formed after 4 days with different concentrations of nitrogen sources is shown. Each treatment included 22–24 roots. Error bars indicate SD. Different letters above the bars indicate significant differences at *P* < 0.01 (Tukey–Kramer test). (B) Phenotypes of NT and mEL5 roots cultured with different nitrogen sources. Rootless shoots were cultured with 10mM (NH_4_)_2_SO_4_, 15mM KNO_3_, or 5mM KNO_2_ for 7 days. Scale bars, 500 µm. (C) Effect of KNO_2_ on LRESs. Preformed roots were treated with 5mM KNO_2_ for 48h. Scale bars, 100 µm. (D) Degree of nitrogen-induced browning at LRESs in NT (white bars) and mEL5 (grey bars). Crown roots were grown for 4 days without nitrogen sources and then were treated with 15mM (NH_4_)_2_SO_4_, 40mM KNO_3_, or 5mM KNO_2_ for 1 day. The average values of 40 shoots per treatment are shown. Error bars indicate SD. Different letters above the bars indicate significant differences at *P* < 0.01 (Tukey–Kramer test with inverse sine transformation).

Root-formation tests were performed using shoots that underwent aseptic hydroponic culture in a low-salt culture solution (LSCS) medium [70 µM CaCl_2_, 36 µM FeSO_4_, 9 µM MnSO_4_, 3 µM ZnSO_4_, 0.2 µM CuSO_4_, 4.5 µM H_3_BO_4_, 0.05 µM Na_2_MoO_4_, 7 µM Na_2_HPO_4_, 16 µM KCl, 150 µM MgCl_2_, 3% (w/v) sucrose, and 10mM MES (pH 5.8)], which is a modification of the nutrient solution reported by Sonoda *et al.* (2003). Husked NT seeds were sterilized in 0.5% (v/v) sodium hypochlorite solution for 30min and then washed five times with distilled water. Seeds were grown in quarter-strength MS medium containing 1% agar at 28 °C under continuous light in a growth chamber until the three-leaf stage. All roots and endosperm tissues were removed and the 12 rootless shoots, whose basal regions were rinsed with distilled water, were placed into holes in a plastic plate that floated on 100ml of LSCS medium with various concentrations of a nitrogen source (Fig. 1A) in a 500ml glass bottle. The shoots were cultured in a growth chamber at 28 °C under continuous light (35 µmol·m^−2^·s^−1^). In tests of mEL5 plants, the subcultured rootless shoots described above were rinsed with distilled water and cultured in LSCS medium in the same way as the rootless NT shoots. After 4 days, the length of the longest crown root of each shoot was measured. This crown root-formation test was repeated twice (total *n* = 22–24 shoots). Statistical analyses (Tukey–Kramer test and Dunnett’s test) were performed using JMP8 software (SAS Institute, Cary, NC, USA).

### Assays of necrosis at lateral-root-emergence sites

**Fig. 4.**
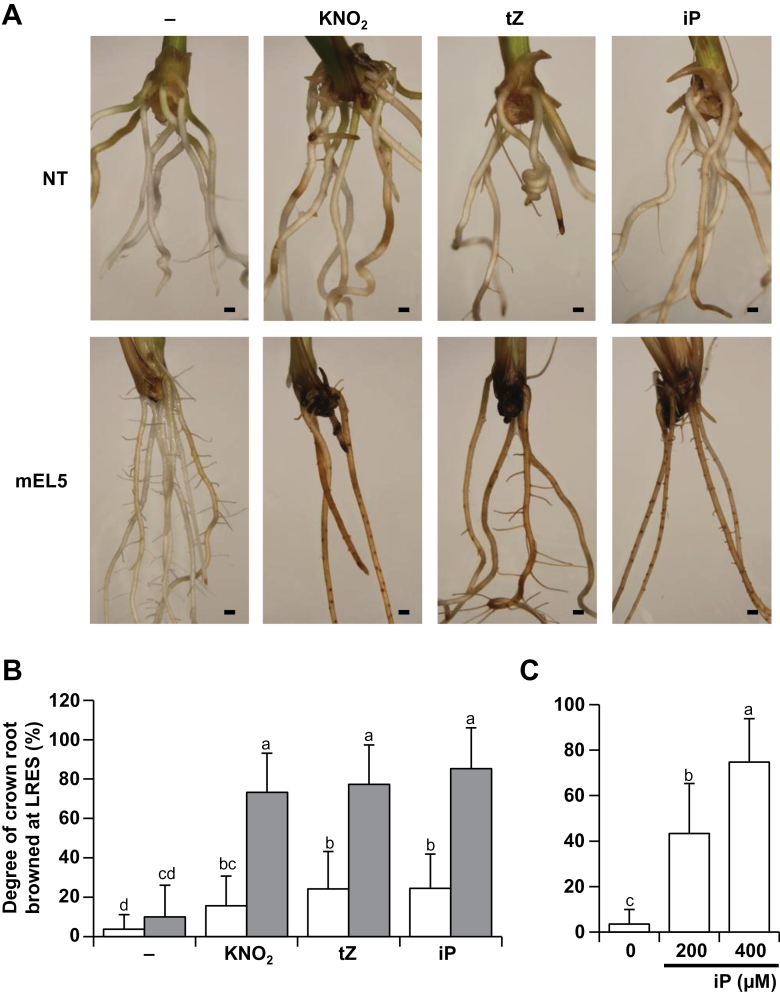
Effect of cytokinin on browning of LRESs. (A) Phenotypes of roots of non-transformants (NT) and transgenic rice plants overexpressing *EL5V162A* (mEL5) treated with 5mM KNO_2_, 200 µM tZ, or 200 µM iP for 3 days. Scale bars, 1mm. (B) Degree of LRES browning in NT (white bars) and mEL5 (grey bars) after treatment with 5mM KNO_2_, 200 µM tZ, or 200 µM iP for 3 days. (C) Degree of LRES browning in NT treated with iP for 2 days. Values are averages (*n* = 40 shoots per treatment) with SD. Different letters above the bars indicate significant differences at *P* < 0.01 (Tukey–Kramer test with inverse sine transformation).

**Fig. 5.**
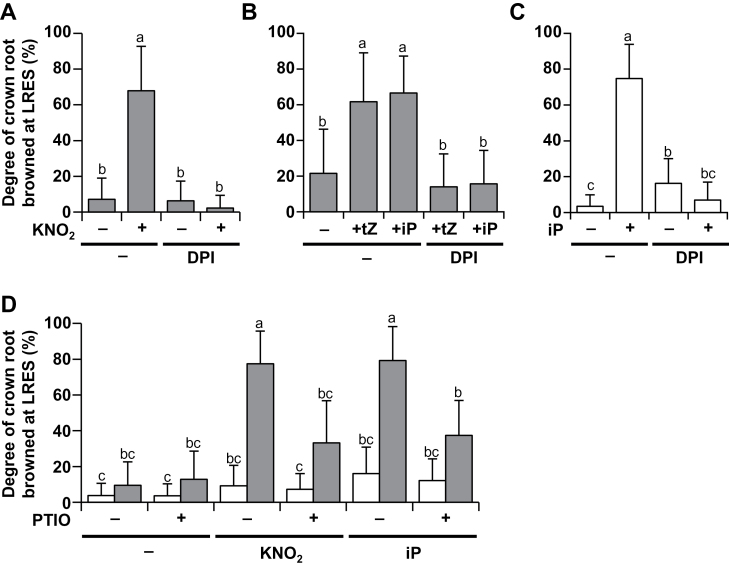
Effects of an inhibitor of superoxide generation and an NO scavenger on browning of LRESs. (A) Degree of LRES browning in mEL5 after treatment with 5mM KNO_2_ and/or 20 µM DPI for 1 day. (B) Degree of LRES browning in mEL5 after treatment with 100 µM tZ or 100 µM iP and/or 20 µM DPI for 2 days. (C) Degree of LRES browning in NT after treatment with 400 µM iP and/or 20 µM DPI for 2 days. (D) Degree of LRES browning in NT (white bars) and mEL5 (grey bars) after treatment with 5mM KNO_2_, 200 µM iP, and/or 100 µM PTIO for 1 day. Roots were grown for 4 days without nitrogen sources and then were simultaneously treated with each chemical. Values are averages (*n* = 40 shoots per treatment) with SD. Different letters above the bars indicate significant differences at *P* < 0.01 (Tukey–Kramer test with inverse sine transformation). NT, non-transformants; mEL5, transgenic rice plants overexpressing *EL5V162A*.

Examination of browning at the lateral-root-emergence site (LRES) was also performed aseptically. Ten rootless shoots per bottle, prepared as mentioned above, were precultured in LSCS medium for 4 days to form crown roots. Roots were then treated with nitrogen and/or cytokinin [*trans*-zeatin (tZ) or *N* ^6^-(2-isopentenyl)-adenine (iP); Nacalai Tesque, Kyoto, Japan] with and without diphenyleneiodonium chloride (DPI) (Toronto Research Chemicals, North York, Canada), or with 2-phenyl-4,4,5,5-tetramethylimidazoline-3-oxide-1-oxyl (PTIO) (Tokyo Chemical Industry Co., Ltd., Tokyo, Japan). Data in Figs 1D, 4B, C, and 5 are presented as the percentage of crown roots per shoot that contained at least one browned LRES. Concentrations of each chemical are indicated in the figure legends. Each assay was repeated four times (total *n* = 40 shoots). Statistical analyses (Tukey–Kramer test and Dunnett’s test) were performed using JMP8 software.

### Microarray analysis

Twelve rootless shoots per bottle were precultured for 4 days in 100ml of LSCS medium to form roots, after which 1mM KNO_2_ was added. After 12h, whole roots were collected from 10 of the 12 treated plants per bottle and were immediately frozen in liquid nitrogen and homogenized using Micro Smash (Tomy Seiko, Tokyo, Japan). Three sets of preparations were repeated for each experimental condition. Total RNA was isolated using an RNeasy Plant Mini Kit (Qiagen, Valencia, CA, USA). Microarray experiments were performed using Rice 4×44 K Microarray RAP-DB (G2519F#15241; Agilent, Santa Clara, CA, USA) as described in Takehisa *et al.* (2012).

The processed raw signal intensity of all probes (45,151) was subjected to 75th-percentile normalization with Subio platform software with the basic plugin (Subio, Kagoshima, Japan) and transformed to a log_2_ scale. A total of 24235 probes (corresponding to 17003 genes) were extracted from 12 microarray data sets after ANOVA normalization. Fold change was calculated as the ratio of log_2_-transformed signal intensity between the untreated NT control and the treatment. We used 1.0 and −1.0 as the critical points for declaring upregulation and downregulation, respectively. Boxplot analysis was performed using Origin 8.6 software (OriginLab, Northampton, MA, USA).

### Quantification of phytohormones

One bottle containing 12 rootless shoots was prepared for each experimental condition and precultured for 4 days in 100ml LSCS medium to form roots. Then, 1mM KNO_2_ was added, and after 6h whole roots from 10 of the 12 treated plants were collected from each bottle and immediately frozen in liquid nitrogen. High-throughput, comprehensive plant-hormone analysis for indole-3-acetic acid (IAA), abscisic acid (ABA), jasmonic acid (JA), jasmonoyl-isoleucine (JA-Ile), salicylic acid (SA), tZ, and iP was performed using liquid chromatography electrospray tandem mass spectrometry (LC-ESI-MS/MS) as described previously (Yoshimoto *et al.*, 2009). A series of these tests was repeated 8–15 times. Statistical analyses of phytohormone contents were performed with the Tukey–Kramer test using JMP8 software.

### Detection of ROS

Nitro blue tetrazolium chloride (NBT; Nacalai Tesque) staining was performed as described by Dunand *et al.* (2007) with modifications. Roots from 20 shoots were prepared for each treatment and the experiments were repeated two times. Ten rootless shoots per bottle were precultured in LSCS medium for 4 days to form roots, and were then treated with 5mM KNO_2_ or 100 µM iP with and without 20 µM DPI for 12h. Roots were collected and floated on 10% Triton X-100 for 5min. After washing twice with 100 µM phosphate buffer (pH 7.4), samples were immersed in 2mM NBT solution (pH 7.4) for 30min.

### Quantitative RT-PCR analysis

**Fig. 7.**
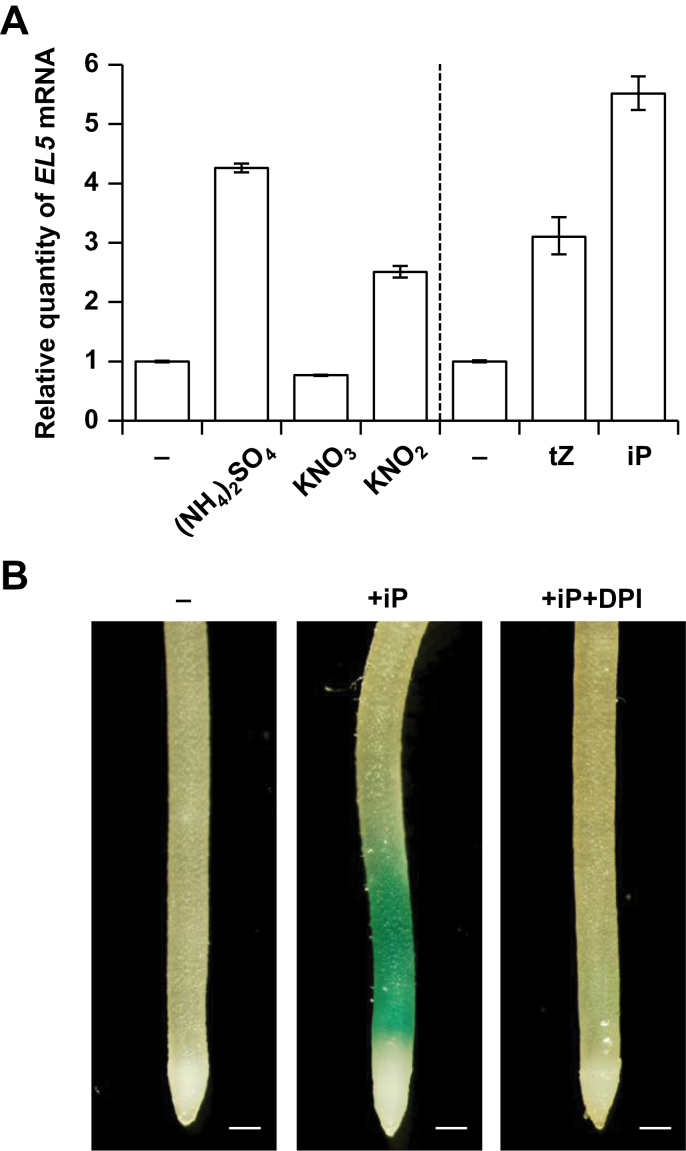
Expression analysis of *EL5*. (A) Quantitative RT-PCR analysis of *EL5* expression in non-transformants. Total RNA was extracted from whole roots treated with 15mM (NH_4_)_2_SO_4_, 40mM KNO_3_, 5mM KNO_2_, 100 µM iP, or 100 µM tZ for 12h. Quantities relative to the *EL5* mRNA level in untreated roots are presented with SE (three technical repeats). (B) GUS staining assay of transgenic rice plants carrying *EL5*p-*GUS*. After rooting for 4 days, crown roots were treated with 100 µM iP with and without 20 µM DPI for 12h. Two independent transgenic lines showed similar results and the representative dataset is presented. Scale bars, 200 µm.

Twelve rootless shoots per bottle were precultured for 4 days in 100ml LSCS to form roots. Whole roots from 10 of the 12 treated plants were collected from each bottle and total RNA was isolated. Trace amounts of DNA were removed by treatment with RQ1 RNase-Free DNase (Promega, Madison, WI, USA); cDNA was prepared from 0.5 µg of total RNA in 10 µl of reaction mixture with a PrimeScript RT reagent Kit (Takara Bio, Otsu, Japan). Quantitative PCR was performed using Mx3000P (Agilent) with 2-µl cDNA solution and a SYBR Premix Ex Taq II Kit (Takara Bio) in 20 µl of reaction mixture. Sets of primers used for amplifying *EL5* and rice ubiquitin gene *rub1* (the control) are shown in Table S1. The initial denaturation step was performed for 10min at 95 °C, followed by cycles of 30 s at 95 °C, 60 s at 55 °C, and 30 s at 72 °C. Relative *EL5* expression was calculated using the 2^−∆∆Ct^ method as described in Livak *et al.* (2001). The experiment was repeated three times, and the representative data are shown in Fig. 7A.

### GUS staining analysis

An *EL5* promoter fragment (2131bp) was amplified from rice genomic DNA using the primers shown in Table S1, and subcloned into pGEM-T vector (Promega). The *EL5* promoter fragment was cloned into the *Nco*I site of a binary vector pSMAHdN627-M2GUS (Hakata *et al.*, 2010). The resulting plasmid carrying *EL5*p-*GUS* was introduced into rice (Nipponbare) via *Agrobacterium*-mediated transformation (Toki *et al.*, 2006). Ten rootless shoots (T_5_ generation) per bottle were precultured in LSCS medium for 4 days to form roots, and were then treated with 100 µM iP with and without 20 µM DPI for 12h. Detached roots were treated with 10% Triton X-100 for 5min and immersed in a solution containing 0.5 mg·ml^−1^ X-Gluc (Nacalai Tesque), 0.5mM potassium ferricyanide, 0.5mM potassium ferrocyanide, and 100mM NaPO_4_ (pH 7.0), and incubated at 37 °C for 24h.

## Results

### Crown root growth of mEL5 is hypersensitive to nitrogen

First, we found that mEL5 (the *EL5V162A*-expressing plants) formed crown roots without necrosis when cultured in nutrient-free conditions. Hydroponic culture of mEL5 shoots in modified MS medium that contained no nitrogen source resulted in crown root formation, indicating that nitrogen is an inhibitory factor in MS media for root formation in mEL5.

To examine the effects of nitrogen sources on crown root formation in mEL5, rootless shoots of mEL5 and NT were aseptically hydrocultured with various concentrations of (NH_4_)_2_SO_4_, KNO_3_, or KNO_2_, and the length of the most elongated crown root was compared. The mEL5 formed crown roots comparable in length to those of NT when cultured without a nitrogen source (Fig. 1A). Although root growth in both mEL5 and NT was inhibited as nitrogen concentration increased, inhibition of mEL5 roots occurred at lower nitrogen concentrations compared to NT (Fig. 1A). On the other hand, the inhibition of crown root elongation by KNO_2_ in transgenic plants overexpressing the wild-type *EL5* was similar to that in NT (Fig. S1).

### Nitrate forms of nitrogen induce more severe necrosis at LRESs in mEL5

Culture of the rootless mEL5 shoots with (NH_4_)_2_SO_4_ diminished crown and lateral root growth, but browning at the LRES did not occur frequently (Fig. 1B). On the other hand, culture with KNO_3_ or KNO_2_ led to browning of the outer cell layers of crown roots at the LRES and prospective LRES, which was followed by termination of lateral root growth. These phenotypes enabled visual evaluation of the response of mEL5 roots to nitrogen, and were almost identical to the phenotype that resulted when mEL5 was cultured in MS medium (Koiwai *et al.*, 2007).

Browning of cells around the LRES and termination of lateral root growth in mEL5 also occurred when the preformed crown roots were treated with KNO_2_ (Fig. 1C). Microscopic observation of mEL5 root sections revealed that cell death accompanied by browning occurred in the meristematic cells of crown and lateral roots in addition to the outer cell layers of crown roots at the LRES (Koiwai *et al.*, 2007). Thus, to describe the effects of treatment with the various chemicals, we considered the number of roots with browned LRES as an indicator of meristematic cell death in the following experiments. The degree of LRES browning in mEL5 was significantly increased by treatment with (NH_4_)_2_SO_4_, KNO_3_, and KNO_2_, with the most severe browning induced by KNO_3_ and KNO_2_ (Fig. 1D). Treatment with KNO_3_ and KNO_2_ resulted in similar phenotypes in mEL5 roots but KNO_2_ led to a clearer phenotype at lower concentrations; thus, we used KNO_2_ to further investigate the function of EL5 in response to nitrogen during root formation. In the analyses that follow, the degree of browning observed in mEL5 roots was regarded as ‘not browned’ (untreated roots) or ‘significantly browned’ (roots treated with 5mM KNO_2_).

### Overexpression of *EL5V162A* affects the expression of nitrite-responsive genes

**Fig. 2.**
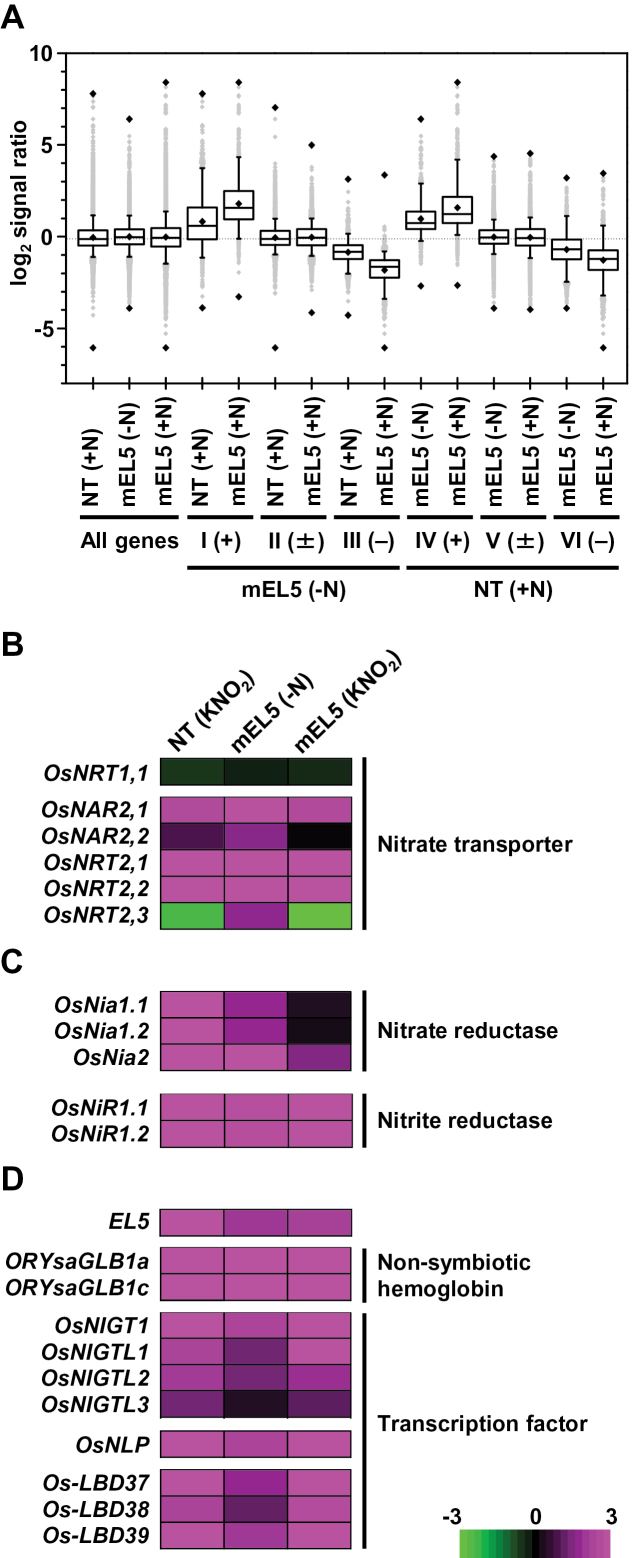
Transcriptome profiling of KNO_2_-treated roots. (A) Boxplot analysis of microarray data (log_2_ signal ratio of values of treated vs untreated non-transformant roots). Boxes represent data between the 25th and 75th percentiles. Whiskers (error bars) above and below the boxes indicate the 90th and 10th percentiles, and black rhombic marks above, within, and below the boxes represent maximum, average, and minimum log_2_ signal ratios, respectively. Lines inside the boxes represent the median. +N, treated with KNO_2_; –N, untreated; +, upregulated (log_2_ signal ratio ≥ 1.0); ±, not changed (between –1.0 and 1.0); −, downregulated (≤ –1.0). I, II, III: fold changes of the 17003 genes in untreated roots of transgenic rice plants overexpressing *EL5V162A* (mEL5) were classified into group I, upregulated genes; group II, unchanged genes; and group III, downregulated genes. IV, V, VI: fold changes of the 17003 genes in KNO_2_-treated non-transformant (NT) roots were classified into group IV, upregulated genes; group V, unchanged genes; and group VI, downregulated genes. (B–D) Gene expression profiles. Expression profile of (B) nitrate transporter genes; (C) nitrogen reductase genes; and (D) nitrogen-induced genes. Data for *EL5* consisted of signals from a probe that specifically detected transcripts of endogenous *EL5*. Magenta and green colours indicate upregulated and downregulated expression, respectively. The colour scale (representing the ratio of the average log_2_ value to the same value of untreated NT) is shown at the bottom right. The RAP-ID and Probe-ID for each gene are listed in Table S2.

To characterize the gene expression in mEL5 roots, we performed microarray analyses of whole roots treated with 1mM KNO_2_ for 12h. For the 17003 genes selected as described in the Materials and methods, the statistical distribution of changes in expression levels compared to untreated NT roots (designated as the fold changes) was analysed using boxplot diagrams (Fig. 2A, Fig. S2A). We first verified that the distribution of fold changes of the 17003 genes was not significantly different among the three data sets: KNO_2_-treated NT and mEL5 roots, and untreated mEL5 roots; the median log_2_ values of the fold changes were nearly zero. Next, we classified the fold changes of the 17003 genes in untreated mEL5 roots into three groups—group I, upregulated genes; group II, unchanged genes; and group III, downregulated genes—and then plotted them according to the fold changes after KNO_2_ treatment of NT and mEL5 roots. The median log_2_ values of the fold changes in group I genes in KNO_2_-treated NT and mEL5 roots increased significantly, while those in group III genes in the same treatments decreased significantly. The expression of these genes was more strongly affected by KNO_2_-treatment in mEL5 roots than in NT roots.

Similarly, we classified the fold changes of the 17003 genes in KNO_2_-treated NT roots into three groups: groups IV, V, and VI (Fig. 2A). The median log_2_ values of the fold changes in group IV and group VI genes increased and decreased significantly, respectively, even in the untreated mEL5 roots, and gene expression was further altered after KNO_2_ treatment. Heat maps with hierarchical clustering of genes that showed changes in expression levels in untreated mEL5 roots (Fig. S2B, C) and in KNO_2_-treated NT roots (Fig. S2D, E) also exhibited overlaps between nitrite-responsive genes in NT roots and genes with altered expression in untreated mEL5 roots.

The overlapped genes included several that code for high-affinity nitrate transporters or nitrogen assimilation, and nitrate-responsive genes including non-symbiotic haemoglobin genes (Ohwaki *et al.*, 2005), but not ammonium transporter genes (Fig. 2B–D, Fig. S3). The endogenous *EL5* was also a nitrite-inducible gene and its expression was elevated in untreated mEL5 roots (Fig. 2D). In addition, KNO_2_ treatment increased the expression of pathogenesis-related genes (Fig. S4) and decreased the expression of photosynthesis-related genes (Fig. S5), most of which changed in mEL5 roots before KNO_2_ treatment. In other words, the expression levels of 73% of nitrite-responsive pathogenesis-related genes (49/67) and 100% of nitrite-responsive photosynthesis-related genes (28/28) were constitutively upregulated and downregulated, respectively, in mEL5. These results indicate that overexpression of *EL5V162A* activates portions of nitrite-signalling pathways that lead to changes in gene expression, and that mEL5 roots retain competence to respond to nitrite.

### Overexpression of *EL5V162A* affects basal levels of phytohormones

**Fig. 3.**
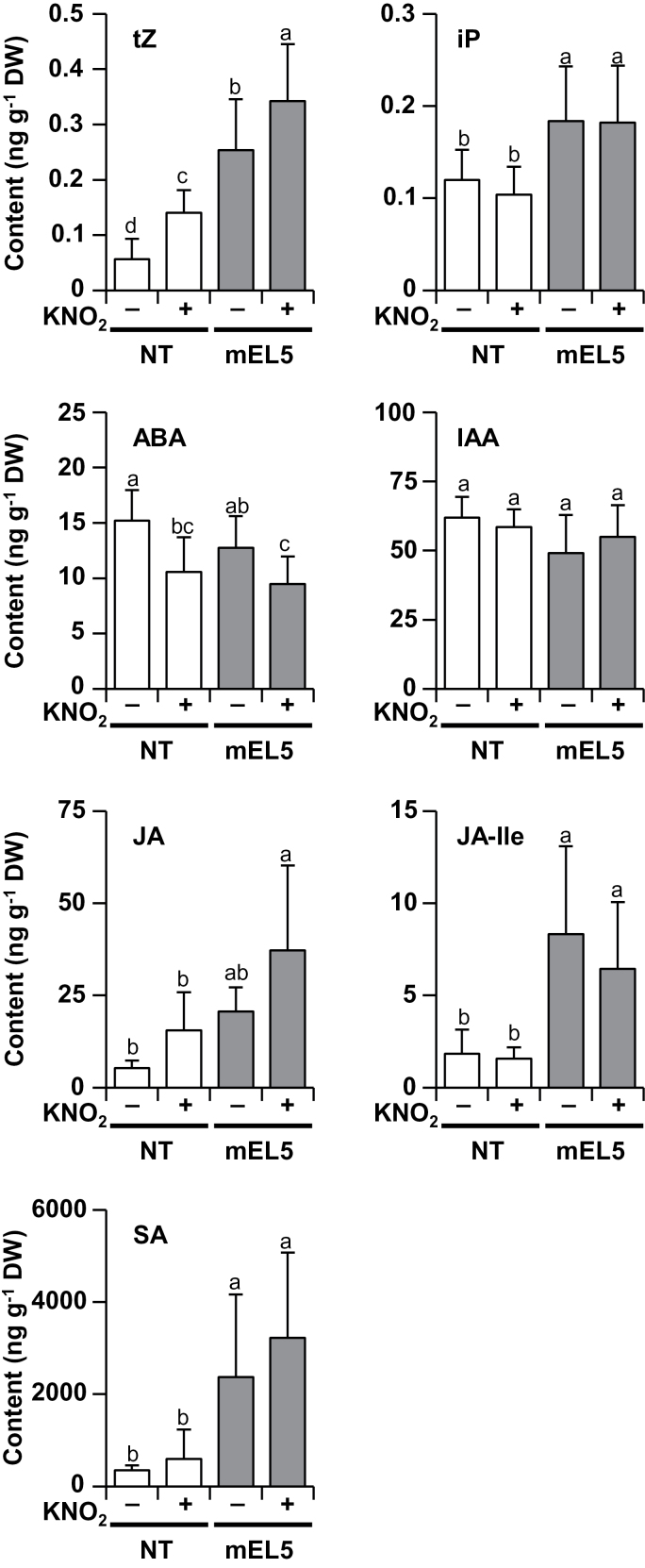
Effect of KNO_2_ treatment and EL5 function on phytohormone contents. Roots were grown for 4 days without nitrogen sources and then treated with 1mM KNO_2_ for 6h. The contents of tZ, iP, ABA, IAA, JA, JA-Ile, and SA in whole roots are presented. Error bars indicate SD. Different letters above the bars indicate significant differences at *P* < 0.05 (*n* = 8–15 independent treatments; Tukey–Kramer test). DW, dry weight; NT, non-transformants; mEL5, transgenic rice plants overexpressing *EL5V162A*.

Next, we quantified the levels of phytohormones in whole roots (Fig. 3). Under KNO_2_ treatment, significant increases and decreases in the contents of tZ and ABA, respectively, were detected. The mEL5 roots contained significantly more tZ compared to NT roots, but ABA content was not affected by *EL5V162A* overexpression. In addition, mEL5 contained significantly more JA, JA-Ile, SA, and iP than NT. IAA content was not affected by either KNO_2_ treatment or by *EL5V162A* overexpression.

Consistent with the phytohormone profile, microarray analysis revealed that KNO_2_ treatment increased the expression of a cytokinin biosynthesis gene (*OsIPT4*), a cytokinin-inactivation gene (*OsCKX3*), and type-A cytokinin-response regulator genes; however, in mEL5 roots, the expression of these genes was elevated prior to KNO_2_ treatment (Fig. S6).

### Excess cytokinin causes cell death at the LRESs

To examine the possibility that the elevated basal level of cytokinin in mEL5 roots caused the LRES browning after KNO_2_ treatment, effects of cytokinin on the induction of browning were examined. Treatment with 200 µM tZ or iP alone induced LRES browning in mEL5 roots to the same extent as treatment with 5mM KNO_2_ alone (Fig. 4A, B). The LRES browning caused by cytokinin was dose-dependent (Fig. S7A). Excess cytokinin also caused LRES browning in NT roots (Fig. 4C). Moreover, concomitant treatment of NT roots with 5mM KNO_2_ and 200 µM tZ or iP significantly increased LRES browning (Fig. S7B). These results indicate that cytokinin is a causal agent of LRES browning in rice.

### Elimination of ROS prevents roots from nitrite- and cytokinin-induced cell death

ROS are widely known to cause cell death in tandem with NO, which is a reduced product of nitrate and nitrite. To clarify the contribution of ROS and NO to nitrite- and cytokinin-induced cell death, we examined the effects of a superoxide-generation inhibitor, DPI, and a NO scavenger, PTIO. Concomitant treatment with 20 µM DPI suppressed LRES browning in mEL5 treated with KNO_2_, tZ, and iP (Fig. 5A, B). DPI also prevented iP-induced browning at the LRES in NT roots (Fig. 5C). Concomitant treatment with 100 µM PTIO also suppressed LRES browning induced by KNO_2_ or iP treatment in mEL5 (Fig. 5D). These results suggest that both ROS and NO are involved in nitrite- and cytokinin-induced cell death.

**Fig. 6.**
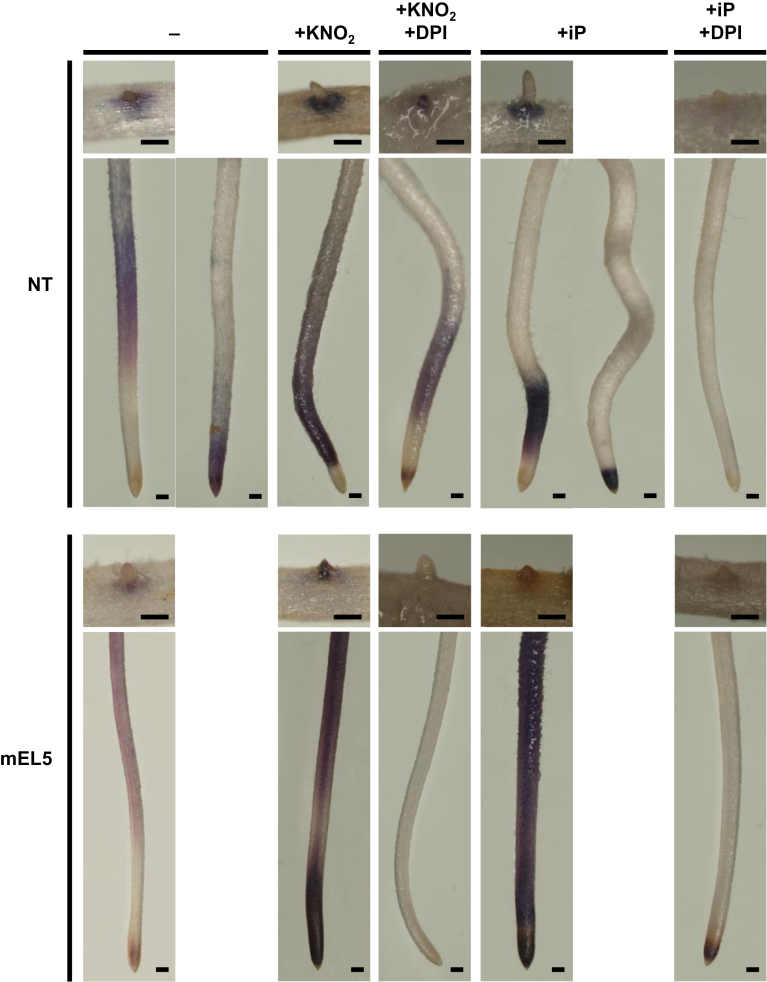
Micrographs of crown roots and LRESs stained with NBT. Roots were grown for 4 days without nitrogen sources and then treated with 1mM KNO_2_ or 100 µM iP with and without 20 µM DPI for 12h. Scale bars, 200 µm. NT, non-transformants; mEL5, transgenic rice plants overexpressing *EL5V162A*.

Next, we observed ROS and NO production in roots to examine the possibility that abnormal production of oxidants may cause nitrite- and cytokinin-induced cell death in mEL5 roots. Accumulation of superoxide as detected with NBT was evident at root tips and LRES of untreated crown roots in NT and mEL5, although two different staining patterns were observed in NT (Fig. 6). Both KNO_2_ and iP treatments increased superoxide accumulation in crown roots, which was inhibited by concomitant treatment with DPI. The mEL5 roots accumulated more superoxide than NT roots, particularly in crown root tips, supporting the hypothesis that ROS are a causal agent of nitrite- and cytokinin-induced cell death in mEL5 roots. The regions that reacted with NBT differed between mEL5 and NT, and they did not necessarily coincide with LRES that were intensely browned by nitrite or cytokinin treatment. On the other hand, KNO_2_ treatment led to NO production, but there was no significant difference between NT and mEL5 in quantity of NO in the culture medium (Fig. S8A) or any evident differences in the localization of NO (Fig. S8B).

### *EL5* expression is induced by nitrogen and cytokinin

We analysed *EL5* expression in NT roots after treatment with various nitrogen sources and cytokinin (Fig. 7A). KNO_2_ and (NH_4_)_2_SO_4_ clearly induced *EL5* expression, and tZ and iP also induced *EL5* expression. GUS-staining analysis of transgenic rice plants carrying *EL5* promoter-*GUS* fusion revealed that GUS activity was induced by iP at the elongation zone of crown roots. The iP-induced GUS staining was suppressed by concomitant treatment with DPI (Fig. 7B), suggesting the involvement of superoxide in the iP-induced *EL5* expression. Induction of GUS activity was not observed after treatment with KNO_2_, probably because KNO_2_ treatment induced a lower level of *EL5* expression (Fig. 7A) as well as superoxide generation in the broader region (Fig. 6) compared to the iP treatment.

## Discussion

### Nitrogen is a causal agent of the rootless phenotype of mEL5

EL5 plays a pivotal role as ubiquitin ligase in crown and lateral root formation by maintaining viability of the RAM (Koiwai *et al.*, 2007). In the present study, we revealed that nitrogen sources, especially nitrate forms, cause RAM death accompanied by browning in plants expressing mutated *EL5*. The mEL5 roots showed increased sensitivity to all nitrogen sources tested, although the location and degree of browning differed according to the nitrogen source (Fig. 1). These differences might be a result of differences in the locus and/or amount of incorporation of the nitrogen form, or the quantity of NO generated after the treatment.

We performed most of the characterizations using nitrite, which produced more rapid and clearer effects than nitrate, but nitrate and nitrite caused similar LRES browning. Similarity in signalling between nitrate and nitrite has been reported in *Arabidopsis*. Transcriptome analysis of *Arabidopsis* roots indicated that nitrite is a more potent signal than nitrate for regulating gene expression, and almost all the pathways and processes induced by nitrate are also induced by nitrite (Wang *et al.*, 2007); Wang *et al.* (2007) thus proposed that the nitrate-sensing system in *Arabidopsis* roots also recognizes nitrite. Our data suggest that nitrate and nitrite act via the same pathway to induce LRES browning.

### Cytokinin-mediated high-nitrogen effects on root formation

Phytohormone profiling of whole roots revealed that mEL5 contains more cytokinin than NT, and nitrite treatment increased cytokinin levels in both mEL5 and NT roots (Fig. 3). Supplementation with nitrate has been shown to induce cytokinin accumulation in the roots of barley (*Hordeum vulgare*) (Samuelson and Larsson, 1993), maize (Sakakibara *et al.*, 1998), *Arabidopsis* (Takei *et al.*, 2001), wheat (*Triticum aestivum*) (Garnica *et al.*, 2010), and rice (Yoshida and Oritani, 1974), indicating that this is a common reaction in plants. Our microarray data suggest that *OsIPT4* is responsible for the increased level of cytokinin in roots by overexpression of *EL5V162A* and in response to nitrite (Fig. S6).

High levels of cytokinin induce apoptosis in plant-cultured cells (Mlejnek and Procházka, 2002; Carimi *et al.*, 2003). We hypothesized that the higher basal levels of cytokinin, which were further increased after nitrite treatment, led to higher sensitivity to nitrogen and thus to RAM death in mEL5. This idea is supported by the finding that mEL5 was hypersensitive to cytokinin compared to NT as reflected in cell death at the LRES, and simultaneous treatment with nitrite and cytokinin also induced LRES browning in NT (Fig. 4, Fig. S7). Our observations are consistent with the finding that overproduction of cytokinin in rice plants by overexpression of *OsIPT* leads to the rootless phenotype (Sakamoto *et al.*, 2006). These results strongly suggest that cytokinin levels should be properly regulated to maintain RAM viability when root formation is altered in response to nitrogen.

We also examined effects of JA and SA on the induction of LRES browning, but, unlike tZ and iP, there were no differences between mEL5 and NT (data not shown). This result suggests that, with the exception of cytokinin, elevated levels of phytohormones in mEL5 are not responsible for the mEL5 phenotype in which the RAM is easily damaged by nitrite. Recently, Novák *et al*. (2013) reported that conditional overproduction of cytokinin by dexamethasone-induced bacterial *ipt* expression in tobacco (*Nicotiana tabacum*) plants caused ROS production in chloroplasts and leaf cell death accompanied by increased levels of JA, SA, and ABA. These researchers speculated that oxidative membrane damage in chloroplasts elevates the content of these stress-related phytohormones (Novák *et al.*, 2013). Likewise, in mEL5 roots, an increase in basal cytokinin levels might have secondary effects on JA and SA levels.

ROS are generated during cytokinin-induced apoptosis in cultured cells of *Arabidopsis* and tobacco (Mlejnek *et al.*, 2003; Carimi *et al.*, 2005), but whether ROS mediates the apoptosis has not been reported. In the present study, we demonstrated that ROS was required for both nitrite- and cytokinin-induced cell death. An NBT staining assay demonstrated that both nitrite and cytokinin treatment increased superoxide accumulation in roots, and this effect was greater in mEL5 roots than in NT roots. These results strongly suggest that increased accumulation of ROS in mEL5 helps to explain the higher sensitivity of mEL5 to nitrite and cytokinin. Therefore, EL5 might function to regulate ROS production under inhibitory nitrogen conditions. In agreement with this proposed function for EL5, the regions in which ROS were detected in NT roots corresponded to the regions in which the expression of *EL5* was induced by cytokinin. *OsPUB15* encodes U-box-type ubiquitin ligase, and its T-DNA insertional mutant, *ospub15*, which contains higher levels of ROS and oxidized proteins, shows a rootless phenotype similar to that of mEL5 (Park *et al.*, 2011). Thus, both OsPUB15 and EL5 probably function to protect the RAM from oxidative damage. However, the steady-state ROS level in *ospub15* was elevated (Park *et al.*, 2011) while that in mEL5 was similar to NT. In addition, the expression level of *OsPUB15* was not affected in mEL5 or by nitrite treatment in our microarray analysis. Therefore, unlike OsPUB15, which is generally involved in tolerance to oxidative damage under conditions including high salt and drought, our data suggest that EL5 plays a role in tolerance to oxidative damage caused by high nitrogen supply.

Carimi *et al.* (2005) reported that NO mediates cytokinin-induced programmed cell death in cultured *Arabidopsis* cells. In tobacco, NO can be produced from nitrite by nitrate reductase and from the root-specific plasma-membrane-bound nitrite-NO reductase (Stöhr *et al.*, 2001). Indeed, we observed NO accumulation after nitrite treatment in the medium cultured with rice roots (Fig. S8A). An NO scavenger suppressed nitrite- and cytokinin-induced cell death in mEL5 (Fig. 5D), indicating the involvement of NO in the cell death. However, there were no significant differences between NT and mEL5 in quantity or location of NO production after nitrite treatment (Fig. S8). Thus, we consider that NO is a trigger of, but not a direct factor in, nitrite- and cytokinin-induced cell death in rice roots; however, we did not detect NO accumulation after cytokinin treatment in our experimental conditions. There was a discrepancy between the loci associated with necrosis and those stained with NBT after nitrite and cytokinin treatment at the LRES. We speculate that other cytotoxic compounds, formed by the reaction with superoxide and/or NO but undetectable by NBT, might execute the LRES cell death.

### EL5 affects the nitrogen-cytokinin signal pathway

Transcriptome profiling of roots revealed that overexpression of *EL5V162A* had a profound effect on gene expression. Boxplot analyses demonstrated that the expression profiles of untreated mEL5 roots significantly resembled those of nitrite-treated NT roots (Fig. 2A, Fig. S2). These results strongly suggest that reduction of EL5 function causes activation of nitrogen signalling despite the absence of a nitrogen source. Recently, transcription factors that regulate nitrogen signalling have been identified in rice and *Arabidopsis* (LOB, Rubin *et al.*, 2009; Albinsky *et al.*, 2010; NIGT1, Sawaki *et al.*, 2013; NLP6 and NLP7, Castaings *et al.*, 2009; Konishi and Yanagisawa, 2013; Marchive *et al.*, 2013). Expression of genes in the same family as these transcription factor genes was induced in rice roots after nitrite treatment, but in mEL5 their expression levels were elevated prior to nitrite treatment (Fig. 2D), which probably contributed to the wide range of alteration in gene expression shown in untreated mEL5 roots.

In contrast with cytokinin, ABA content was deduced after nitrite treatment (Fig. 3). Generally, ABA signalling is mediated by ROS and NO (Gayatri *et al.*, 2013). Thus, it is possible that the reduction of ABA content is an upstream event in the nitrite-cytokinin signal pathway. However, basal levels of ABA-related gene expression, ABA content, and their alteration patterns after nitrite treatment were similar in mEL5 and NT roots (Fig. 3, Fig. S9). We also examined the effect of ABA on the induction of LRES browning in mEL5; simultaneous treatment with nitrite and ABA did not negate the browning (data not shown). In addition, the expression of *EL5* was induced by cytokinin but not by ABA (Koiwai *et al.*, 2007). These facts suggest that the pathway leading to the reduction of ABA content is independent of nitrite-induced cytokinin synthesis.

**Fig. 8.**
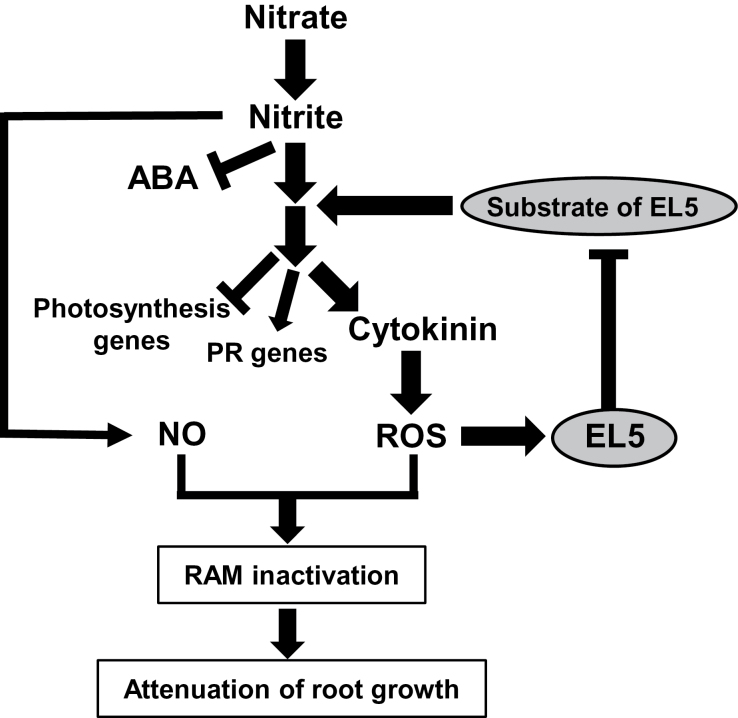
Model of the effects of high concentrations of nitrogen on root growth and EL5 function, derived from the present comparative studies between non-transformants and transgenic rice plants overexpressing *EL5V162A* (mEL5). PR genes, pathogenesis-related genes.

Taken together, these data strongly suggest that EL5 acts on the nitrogen-signalling pathway(s) leading to cytokinin synthesis, transcriptional induction of nitrogen-related and pathogenesis-related genes, and suppression of photosynthesis-related gene expression; the data also suggest that EL5 does not act on the pathway leading to suppression of ABA synthesis. Because EL5 is involved in root formation as ubiquitin ligase (Koiwai *et al.*, 2007), we conclude that EL5 affects the nitrate-cytokinin signal pathway via ubiquitination of the putative target protein, which leads to alteration of RAM vigour through ROS generation (Fig. 8). Identification of the target protein would elucidate the biological significance and molecular mechanism of its additive action to the effects of nitrogen.

## Supplementary material

Supplementary material is available at *JXB* online.

Supplementary Fig. S1. Inhibitory effect of nitrogen on crown root elongation in transgenic rice plants overexpressing *EL5*.

Supplementary Fig. S2. Effect of KNO_2_ treatment and EL5 function on gene expression in whole roots.

Supplementary Fig. S3. Expression profiles of representative nitrogen-related genes.

Supplementary Fig. S4. Expression profiles of representative pathogenesis-related genes.

Supplementary Fig. S5. Expression profiles of representative photosynthesis-related genes.

Supplementary Fig. S6. Expression profiles of representative genes for biosynthesis, inactivation, and response of cytokinin.

Supplementary Fig. S7. Effect of cytokinin on browning at lateral root emergence sites (LRES).

Supplementary Fig. S8. Accumulation of NO after nitrite treatment.

Supplementary Fig. S9. Expression profiles of representative abscisic acid (ABA)-related genes.

Supplementary Table S1. Oligonucleotide primers used in this work.

Supplementary Table S2. RAP-ID and Probe-ID list of the nitrogen-related genes shown in Fig. 2.

## Funding

This work was supported by Grants-in-Aids for Foundation Research (B) from the Ministry of Education, Culture, Sports, Science and Technology, Japan [grant number 20380030], and by a grant from the Japan Society for the Promotion of Science initiated by the Council for Science and Technology Policy [grant number GS028].

## Supplementary Material

Supplementary Data

## Abbreviations:

ABA: abscisic acid
ATL: Arabidopsis Tóxicos en Levadura
DPI: diphenyleneiodonium chloride
IAA: indole-3-acetic acid
iP: *N* ^6^-(2-isopentenyl)-adenine
JA: jasmonic acid
JA-Ile: jasmonoyl-isoleucine
LRES: lateral-root-emergence site
LSCS: low-salt culture solution
MS medium: Murashige and Skoog medium
NBT: nitro blue tetrazolium chloride
NT: non-transgenic
PTIO: 2-phenyl-4,4,5,5-tetramethylimidazoline-3-oxide-1-oxyl
RAM: root apical meristem
ROS: reactive oxygen species
SA: salicylic acid
tZ: *trans*-zeatin.
