# Supplementary material for: Ubiquitin ligase EL5 maintains the viability of root meristems by influencing cytokinin-mediated nitrogen effects in rice
Source: J Exp Bot. 2014 Mar 24;65(9):2307–18. doi: 10.1093/jxb/eru110 (PMC4036501; doi:10.1093/jxb/eru110)
Supplement: Supplementary Data [file supp_65_9_2307__index.html]

Ubiquitin ligase EL5 maintains the viability of root meristems by influencing cytokinin-mediated nitrogen effects in rice — Ubiquitin ligase EL5 maintains the viability of root meristems by influencing cytokinin-mediated nitrogen effects in rice — Supplementary Data 

# Ubiquitin ligase EL5 maintains the viability of root meristems by influencing cytokinin-mediated nitrogen effects in rice

## Supplementary Data

Data files

**Files in this Data Supplement:**

- Supplementary Data - Supplementary Data
