# Supplementary material for: Ubiquitin ligase EL5 maintains the viability of root meristems by influencing cytokinin-mediated nitrogen effects in rice
Source: J Exp Bot. 2014 Mar 24;65(9):2307–18. doi: 10.1093/jxb/eru110 (PMC4036501; doi:10.1093/jxb/eru110)
Supplement: Supplementary Data [file supp_eru110_jexbot115568_file001.pdf]

Journal of Experimental Botany

Ubiquitin ligase EL5 maintains the viability of root meristems by influencing cytokinin-mediated nitrogen effects in rice

Susumu Mochizuki, Yusuke Jikumaru, Hidemitsu Nakamura, Hanae Koiwai, Keisuke Sasaki, Yuji Kamiya, Hiroaki Ichikawa, Eiichi Minami, Yoko Nishizawa

Supporting Information

Supplementary Table S1. Oligonucleotide primers used in this work.

Supplementary Table S2. RAP-ID and Probe-ID list of the nitrogen-related genes shown in Fig. 2.

Supplementary Fig. S1. Inhibitory effect of nitrogen on crown root elongation in transgenic rice plants overexpressing *EL5*.
Length of the longest crown root of non-transformants (NT, white bars) and two independent transgenic lines overexpressing the wild-type *EL5* (gray bars) formed after 4 d under different concentrations of KNO_2. Values are averages (n = 22 to 23 roots per treatment) with SD. Different letters above the bars indicate significant differences at $P < 0.01$ (Tukey-Kramer test).

Supplementary Fig. S2. Effect of KNO_2 treatment and EL5 function on gene expression in whole roots.
(A) Values of the box-plot analysis shown in Fig. 2A.
(B, C) Heat maps with hierarchical clustering of the expression levels of up-regulated genes (group I) and down-regulated genes (group III) in untreated roots of transgenic rice plants overexpressing *EL5V162A* (mEL5).
(D, E) Heat maps with hierarchical clustering of up-regulated genes (group IV) and down-regulated genes (group VI) in KNO_2-treated non-transformants (NT).
Magenta and green colors indicate up- and down-regulated expression, respectively. The color scale (representing the ratio of the average $\log_2$ value to the same value of untreated NT) is shown at the bottom right.

Supplementary Fig. S3. Expression profiles of representative nitrogen-related genes.
Magenta and green colors indicate up- and down-regulated expression respectively. The color scale (representing the ratio of the average $\log_2$ value to the same value of untreated non-transformants) is shown at the bottom. NT, non-transformants; mEL5, transgenic rice plants overexpressing *EL5V162A*.

Supplementary Fig. S4. Expression profiles of representative pathogenesis-related genes.

Magenta and green colors indicate up- and down-regulated expression respectively. The color scale (representing the ratio of the average log_2 value to the same value of untreated non-transformants) is shown at the bottom right. NT, non-transformants; mEL5, transgenic rice plants overexpressing *EL5V162A*.

Supplementary Fig. S5. Expression profiles of representative photosynthesis-related genes.

Magenta and green colors indicate up- and down-regulated expression respectively. The color scale (representing the ratio of the average log_2 value to the same value of untreated non-transformants) is shown at the bottom. NT, non-transformants; mEL5, transgenic rice plants overexpressing *EL5V162A*.

Supplementary Fig. S6. Expression profiles of representative genes for biosynthesis, inactivation, and response of cytokinin.

Magenta and green colors indicate up- and down-regulated expression respectively. The color scale (representing the ratio of the average log_2 value to the same value of untreated non-transformants) is shown at the bottom. NT, non-transformants; mEL5, transgenic rice plants overexpressing *EL5V162A*.

Supplementary Fig. S7. Effect of cytokinin on browning at lateral root emergence sites (LRES).

(A) Dose-dependency of cytokinin-induced LRES browning. Roots were grown for 4 d without nitrogen sources and then treated with different concentrations of iP for 2 d.

(B) Effect of cytokinin on nitrite-induced LRES browning in non-transformants (NT). Roots were grown for 4 d without nitrogen sources and then treated with 200 µM tZ or iP with or without 5 mM KNO_2 for 3 d.

Values are averages (n = 40 shoots per treatment) with SD. White bars, NT; gray bars, transgenic rice plants overexpressing *EL5V162A* (mEL5). Different letters above the bars indicate significant differences at $P < 0.01$ (Tukey-Kramer test with inverse sine transformation).

Supplementary Fig. S8. Accumulation of NO after nitrite treatment.

(A) NO accumulation after KNO_2 treatment in non-transformants (NT, white bars) and transgenic rice plants overexpressing *EL5V162A* (mEL5, gray bars). Roots from 10 shoots per bottle were grown for 4 d without nitrogen sources, and then 10 µL DAF2-DA solution (1.82 g L^{-1} in DMSO) was added simultaneously with or without 5 mM KNO_2 for 1 d. Quantities of DAF2-T fluorescence in 100 µL culture medium from three independent bottles were measured, and average strength of fluorescence is presented. Error bars indicate SD. Different letters above the bars indicate significant differences at $P < 0.01$ (Tukey-Kramer test with inverse sine transformation).

(B) NO accumulation at lateral root emergence sites (LRES) after KNO_2 treatment. Typical confocal micrographs of DAF2-T fluorescence at the LRES are presented with each bright field image. Roots were grown for 4 d without nitrogen sources and then treated with or without 1 mM KNO_2 for 1 d with DAF2-DA (final concentration 0.182 mg L^{-1}). The fluorescence-field panels are confocal images composed of stacked Z-series images. Scale bars = 100 µm. Images were observed by TCS SP5 confocal microscope (Leica, Wetzlar, Germany). DAF2-T fluorescence was excited at 488-nm, and detected at 500–540 nm. Images were processed and arranged by LAS AF software (Leica), MetaMorph software (Molecular Devices, Inc., Sunnyvale, CA), and Photoshop CS4 (Adobe, San Jose, CA).

Supplementary Fig. S9. Expression profiles of representative abscisic acid (ABA)-related genes.

Magenta and green colors indicate up- and down-regulated expression respectively. The color scale (representing the ratio of the average $\log_2$ value to the same value of untreated non-transformant) is shown at the bottom. NT, non-transformants; mEL5, transgenic rice plants overexpressing *EL5V162A*.

Supplementary Table S1. Oligonucleotide primers used in this work.

Primer name	Sequence (5′ to 3′)	Target gene (GenBank ID)
**<u>Primers for qRT-PCR</u>**		
EL5-QRT-FW4	GCGAGGTCGTCAAGTATTATAAGG	*EL5* (AB045120)
EL5-QRT-RV4	CCATCCATACATTTCTCTTTGCTAC	
rub1-sense	CCAGTAAGTCCTCAGCCATGGAG	*UBQ* (ubiquitin; AK121590)
rub1-antisense	GGACACAATGATTAGGGAATCAC	
**<u>Primers for construction of *EL5*p-*GUS* plasmid</u>**		
*EL5*p-sense	GCAAATAGGGTCTCTCGTTAGC	*EL5* promoter fragment
*EL5*p-antisense	GACGAAGACTCGTCCATGGC	

Supplementary Table S2. RAP-ID and Probe-ID list of the nitrogen-related genes shown in Fig. 2.

Genes	RAP-ID	Probe-ID
OsNRT1,1	Os03g0235900	Os03g0235900|mRNA|AF140606|CDS+3'UTR
OsNAR2.1	Os02g0595900	Os02g0595900|COMBINER_EST|CI554982|0
OsNAR2.2	Os04g0480200	Os04g0480200|mRNA|AK109571|CDS+3'UTR
OsNRT2.1	Os02g0112100	Os02g0112100|mRNA|AB008519|CDS+3'UTR
OsNRT2.2	Os02g0112600	Os02g0112600|mRNA|AK109733|CDS+3'UTR
OsNRT2.3	Os01g0704100	Os01g0704100|mRNA|AK109776|CDS+3'UTR
OsNia1.1	Os08g0468100	Os08g0468100|mRNA|AK101662|CDS+3'UTR
OsNia1.2	Os08g0468700	Os08g0468700|COMBINER_EST|CI196459|6
OsNia2	Os02g0770800	Os02g0770800|mRNA|AK102178|CDS+3'UTR
OsNiR1.1	Os01g0357100	Os01g0357100|mRNA|AK061893|CDS+3'UTR
OsNiR1.2	Os02g0765900	Os02g0765900|COMBINER_EST|CI138324|6
EL5	Os02g0559800 – Os02g0561800	Os02g0559800|mRNA|AB045120|CDS+3'UTR[1)]
ORYsaGLB1a	Os03g0233900	Os03g0233900|mRNA|AK064054|CDS+3'UTR
ORYsaGLB1c	Os03g0234000	Os03g0234000|COMBINER_EST|Os03g0234000|8
OsNIGT1	Os02g0325600	Os02g0325600|mRNA|AK101809|CDS+3'UTR
OsNIGTL1	Os03g0764600	Os03g0764600|mRNA|AK105625|CDS+3'UTR
OsNIGTL2	Os07g0119300	Os07g0119300|mRNA|AK070421|CDS+3'UTR
OsNIGTL3	Os12g0586300	Os12g0586300|COMBINER_EST|Os12g0586300|8
OsNLP	Os02g0136000	Os02g0136000|COMBINER_EST|CI549716|3
Os-LBD37	Os03g0445700	Os03g0445700|mRNA|AK071624|CDS+3'UTR
Os-LBD38	Os07g0589000	Os07g0589000|mRNA|AK069813|CDS+3'UTR
Os-LBD39	Os03g0609500	Os03g0609500|COMBINER_EST|CI261705|6

1) This probe can hybridize to the 3′UTR region (148-bp from the 3′ end of the coding sequence) of mRNA, which is transcribed from 6 copies of rice genes (Os02g0559800 to Os02g0561800).

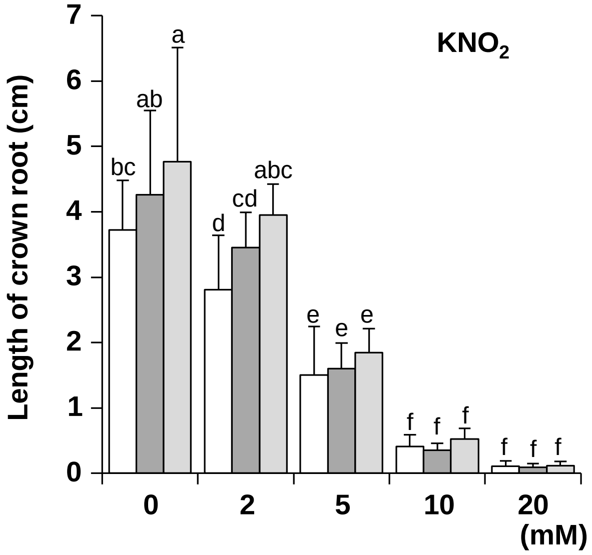
KNO2
Length of crown root (cm)
7
6
5
4
3
2
1
0
a
ab
bc
abc
cd
d
e
e
e
f
f
f
f
f
f
0
2
5
10
20
(mM)

Figure S1

A

		Genotype (treatment)	*N*	Average	SD	Min	10%	25%	Median	75%	90%	Max	75%-25%
All genes		NT(KNO2)	17003	-0.04850	0.00602	-6.05327	-0.85608	-0.49241	-0.14233	0.33762	0.80579	7.78790	0.83003
		mEL5(mock)	17003	-0.00032	0.00602	-3.90720	-0.78173	-0.40755	-0.03281	0.39869	0.78713	6.40276	0.80624
		mEL5(KNO2)	17003	-0.02660	0.00602	-6.05654	-1.04595	-0.53804	-0.06921	0.47090	0.93821	8.41384	1.00894
mEL5 (mock)	+1.0 ≤ (I)	NT(KNO2)	1084	0.82540	0.02383	-3.87858	-0.79610	-0.15314	0.58852	1.58829	2.77305	7.78790	1.74143
		mEL5(KNO2)	1084	1.79530	0.02383	-3.27832	0.38527	0.94482	1.57090	2.49804	3.54988	8.41384	1.55322
	-1.0 to +1.0 (II)	NT(KNO2)	14859	-0.05520	0.00644	-6.05327	-0.75613	-0.45136	-0.12771	0.31880	0.72655	7.03442	0.77016
		mEL5(KNO2)	14859	-0.03170	0.00644	-4.14735	-0.83143	-0.47426	-0.06321	0.41490	0.78276	4.98417	0.88916
	≤ -1.0 (III)	NT(KNO2)	1060	-0.84880	0.02410	-4.28122	-1.66908	-1.22111	-0.82279	-0.46328	-0.10256	3.13055	0.75783
		mEL5(KNO2)	1060	-1.81770	0.02410	-6.05654	-2.89847	-2.23606	-1.64985	-1.28225	-1.01272	3.35848	0.95381
NT (KNO_2)	+1.0 ≤ (IV)	mEL5(mock)	1148	0.97070	0.02316	-2.68527	0.06895	0.40675	0.74568	1.35755	2.19755	6.40276	0.95080
		mEL5(KNO2)	1148	1.57590	0.02316	-2.65615	0.35494	0.74063	1.22557	2.17110	3.40818	8.41384	1.43047
	-1.0 to +1.0 (V)	mEL5(mock)	14688	-0.02050	0.00648	-3.89922	-0.70011	-0.38762	-0.04644	0.35287	0.68903	4.36952	0.74049
		mEL5(KNO2)	14688	-0.05190	0.00648	-3.97613	-0.88220	-0.48810	-0.07618	0.40600	0.78343	4.53773	0.89410
	≤ -1.0 (VI)	mEL5(mock)	1167	-0.70180	0.02297	-3.90720	-2.10099	-1.23904	-0.68503	-0.15941	0.56429	3.20129	1.07963
		mEL5(KNO2)	1167	-1.28490	0.02297	-6.05654	-2.71745	-1.80970	-1.21681	-0.74917	-0.06232	3.45849	1.06053

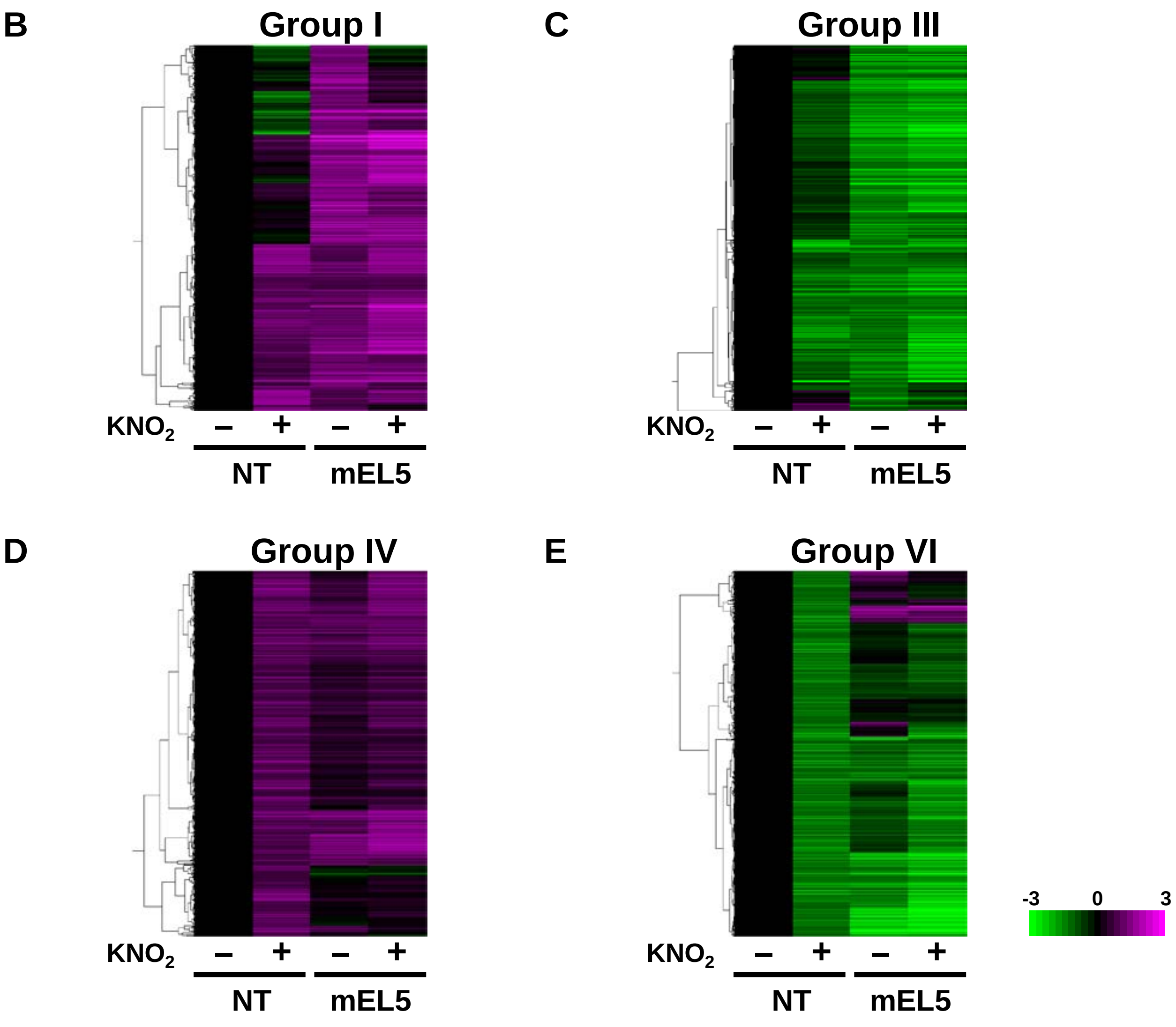

Figure S2

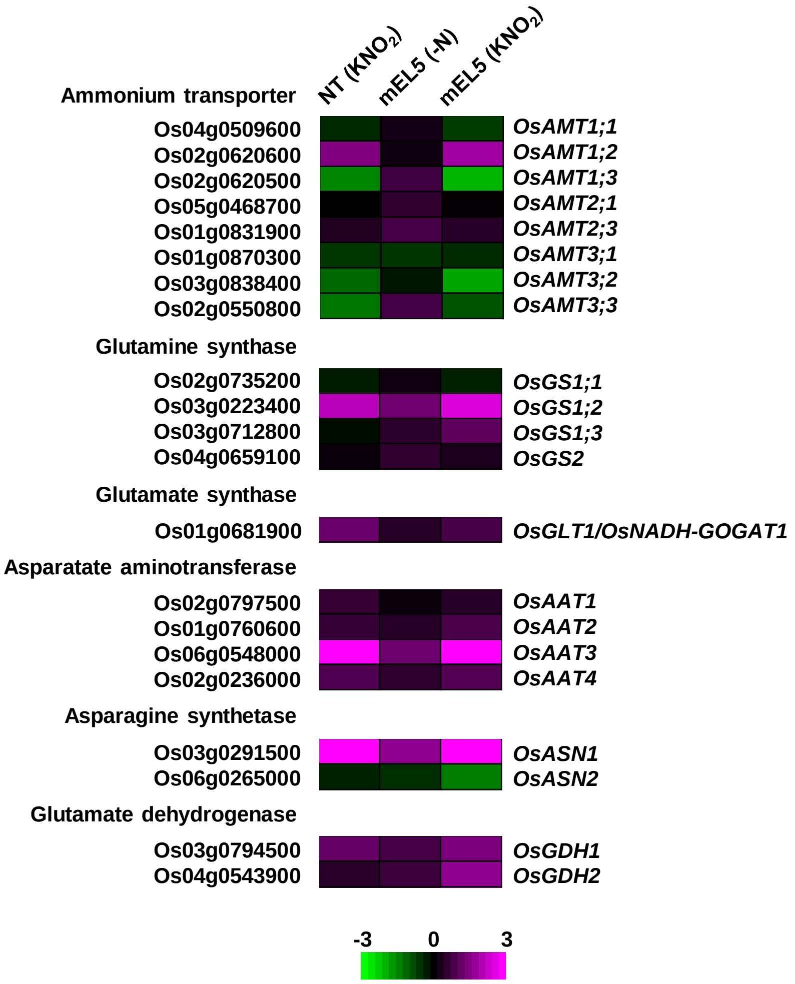

NT (KNO2)
mEL5 (-N)
mEL5 (KNO2)
Ammonium transporter
Os04g0509600
Os02g0620600
Os02g0620500
Os05g0468700
Os01g0831900
Os01g0870300
Os03g0838400
Os02g0550800
OsAMT1;1
OsAMT1;2
OsAMT1;3
OsAMT2;1
OsAMT2;3
OsAMT3;1
OsAMT3;2
OsAMT3;3
Glutamine synthase
Os02g0735200
Os03g0223400
Os03g0712800
Os04g0659100
OsGS1;1
OsGS1;2
OsGS1;3
OsGS2
Glutamate synthase
Os01g0681900
OsGLT1/OsNADH-GOGAT1
Asparatate aminotransferase
Os02g0797500
Os01g0760600
Os06g0548000
Os02g0236000
OsAAT1
OsAAT2
OsAAT3
OsAAT4
Asparagine synthetase
Os03g0291500
Os06g0265000
OsASN1
OsASN2
Glutamate dehydrogenase
Os03g0794500
Os04g0543900
OsGDH1
OsGDH2
-3
0
3

Figure S3

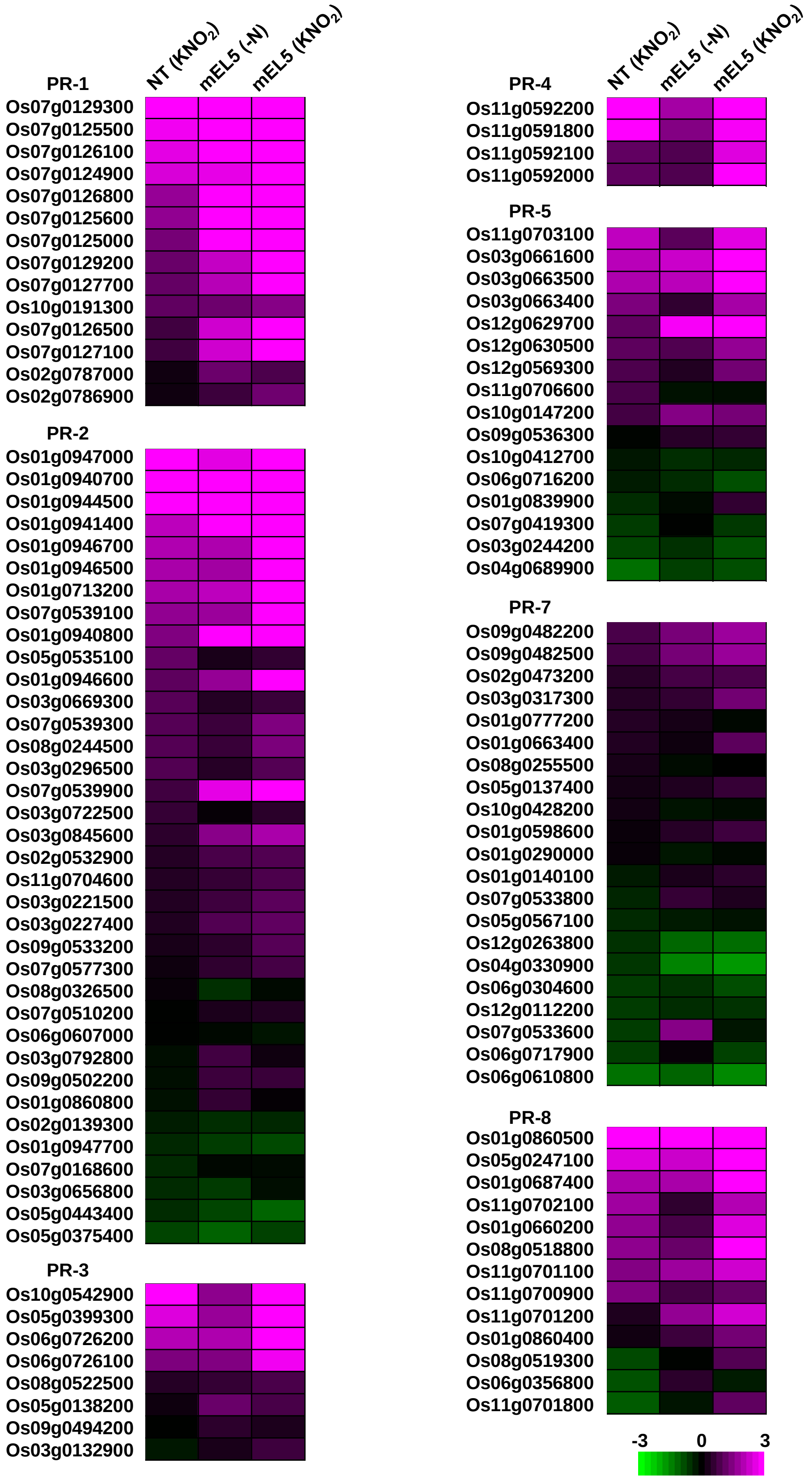
NT (KNO_2)
mEL5 (-N)
mEL5 (KNO_2)
PR-1
Os07g0129300
Os07g0125500
Os07g0126100
Os07g0124900
Os07g0126800
Os07g0125600
Os07g0125000
Os07g0129200
Os07g0127700
Os10g0191300
Os07g0126500
Os07g0127100
Os02g0787000
Os02g0786900
PR-2
Os01g0947000
Os01g0940700
Os01g0944500
Os01g0941400
Os01g0946700
Os01g0946500
Os01g0713200
Os07g0539100
Os01g0940800
Os05g0535100
Os01g0946600
Os03g0669300
Os07g0539300
Os08g0244500
Os03g0296500
Os07g0539900
Os03g0722500
Os03g0845600
Os02g0532900
Os11g0704600
Os03g0221500
Os03g0227400
Os09g0533200
Os07g0577300
Os08g0326500
Os07g0510200
Os06g0607000
Os03g0792800
Os09g0502200
Os01g0860800
Os02g0139300
Os01g0947700
Os07g0168600
Os03g0656800
Os05g0443400
Os05g0375400
PR-3
Os10g0542900
Os05g0399300
Os06g0726200
Os06g0726100
Os08g0522500
Os05g0138200
Os09g0494200
Os03g0132900
PR-4
Os11g0592200
Os11g0591800
Os11g0592100
Os11g0592000
PR-5
Os11g0703100
Os03g0661600
Os03g0663500
Os03g0663400
Os12g0629700
Os12g0630500
Os12g0569300
Os11g0706600
Os10g0147200
Os09g0536300
Os10g0412700
Os06g0716200
Os01g0839900
Os07g0419300
Os03g0244200
Os04g0689900
PR-7
Os09g0482200
Os09g0482500
Os02g0473200
Os03g0317300
Os01g0777200
Os01g0663400
Os08g0255500
Os05g0137400
Os10g0428200
Os01g0598600
Os01g0290000
Os01g0140100
Os07g0533800
Os05g0567100
Os12g0263800
Os04g0330900
Os06g0304600
Os12g0112200
Os07g0533600
Os06g0717900
Os06g0610800
PR-8
Os01g0860500
Os05g0247100
Os01g0687400
Os11g0702100
Os01g0660200
Os08g0518800
Os11g0701100
Os11g0700900
Os11g0701200
Os01g0860400
Os08g0519300
Os06g0356800
Os11g0701800
-3
0
3

Figure S4

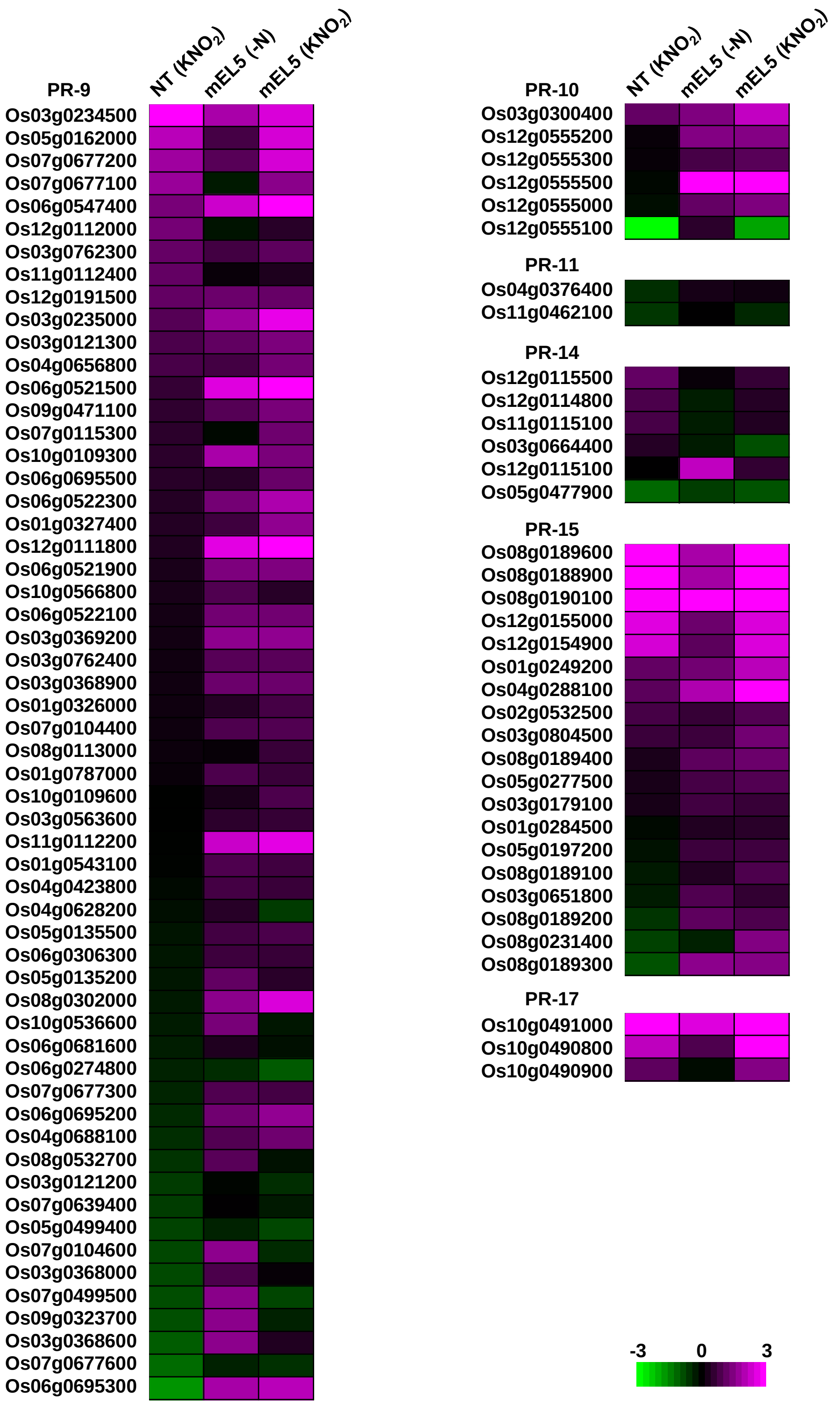

Figure S4 (continued)

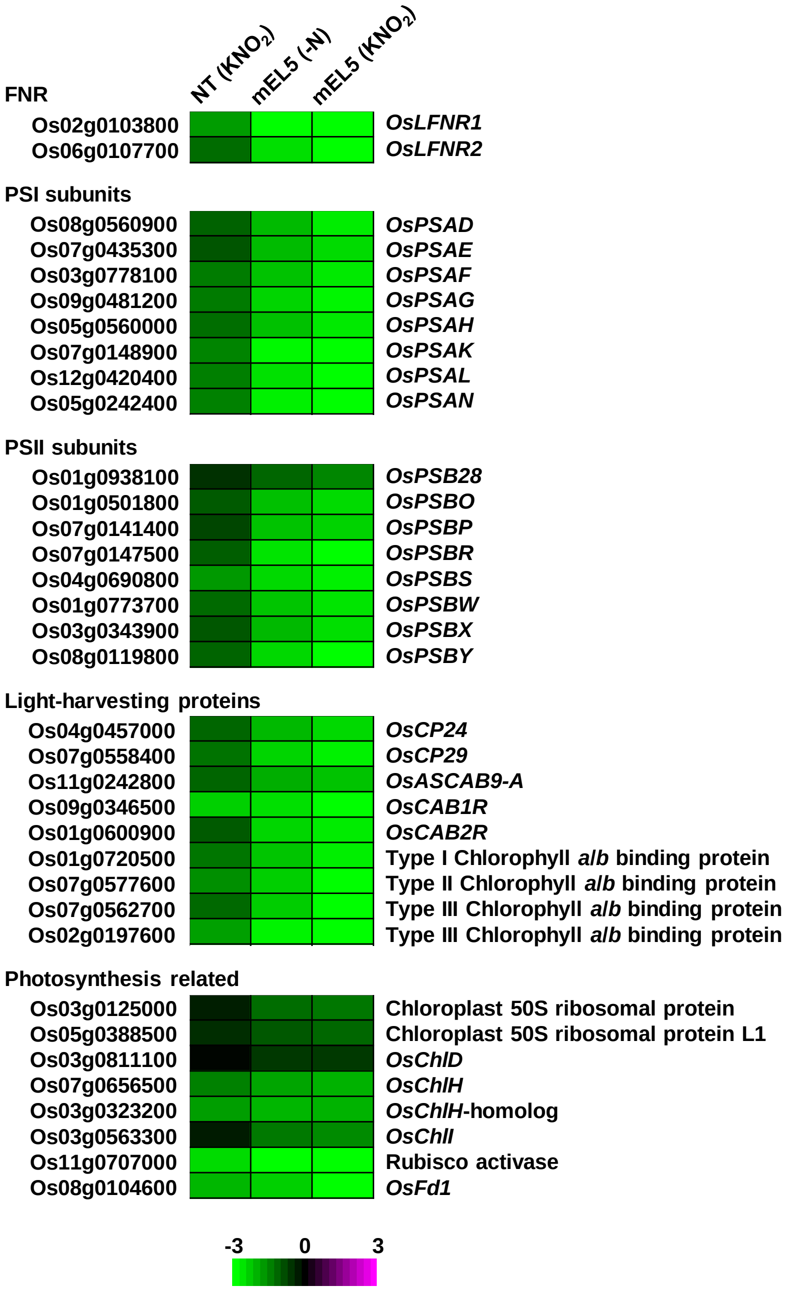
NT (KNO2)
mEL5 (-N)
mEL5 (KNO2)
FNR
Os02g0103800
Os06g0107700
OsLFNR1
OsLFNR2
PSI subunits
Os08g0560900
Os07g0435300
Os03g0778100
Os09g0481200
Os05g0560000
Os07g0148900
Os12g0420400
Os05g0242400
OsPSAD
OsPSAE
OsPSAF
OsPSAG
OsPSAH
OsPSAK
OsPSAL
OsPSAN
PSII subunits
Os01g0938100
Os01g0501800
Os07g0141400
Os07g0147500
Os04g0690800
Os01g0773700
Os03g0343900
Os08g0119800
OsPSB28
OsPSBO
OsPSBP
OsPSBR
OsPSBS
OsPSBW
OsPSBX
OsPSBY
Light-harvesting proteins
Os04g0457000
Os07g0558400
Os11g0242800
Os09g0346500
Os01g0600900
Os01g0720500
Os07g0577600
Os07g0562700
Os02g0197600
OsCP24
OsCP29
OsASCAB9-A
OsCAB1R
OsCAB2R
Type I Chlorophyll a/b binding protein
Type II Chlorophyll a/b binding protein
Type III Chlorophyll a/b binding protein
Type III Chlorophyll a/b binding protein
Photosynthesis related
Os03g0125000
Os05g0388500
Os03g0811100
Os07g0656500
Os03g0323200
Os03g0563300
Os11g0707000
Os08g0104600
Chloroplast 50S ribosomal protein
Chloroplast 50S ribosomal protein L1
OsChlD
OsChlH
OsChlH-homolog
OsChlI
Rubisco activase
OsFd1
-3
0
3

Figure S5

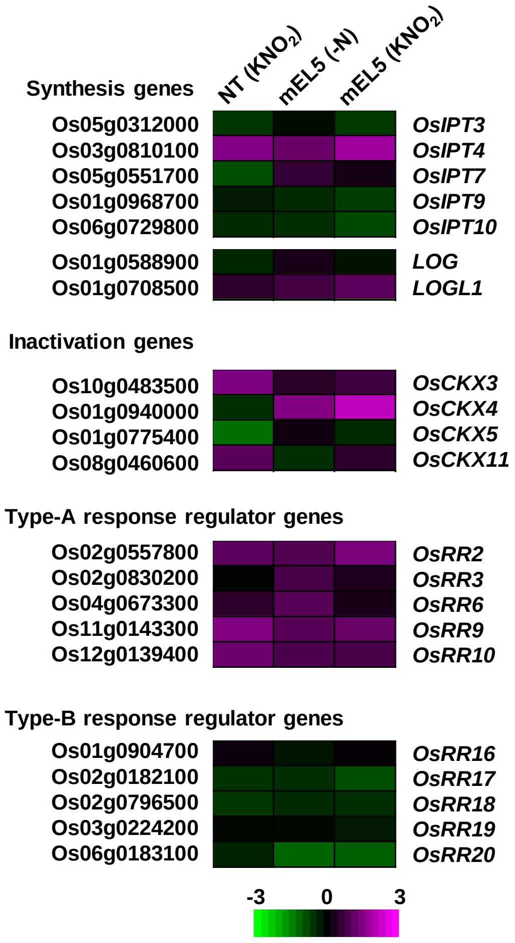

NT (KNO2)
mEL5 (-N)
mEL5 (KNO2)
Synthesis genes
Os05g0312000 OsIPT3
Os03g0810100 OsIPT4
Os05g0551700 OsIPT7
Os01g0968700 OsIPT9
Os06g0729800 OsIPT10
Os01g0588900 LOG
Os01g0708500 LOGL1
Inactivation genes
Os10g0483500 OsCKX3
Os01g0940000 OsCKX4
Os01g0775400 OsCKX5
Os08g0460600 OsCKX11
Type-A response regulator genes
Os02g0557800 OsRR2
Os02g0830200 OsRR3
Os04g0673300 OsRR6
Os11g0143300 OsRR9
Os12g0139400 OsRR10
Type-B response regulator genes
Os01g0904700 OsRR16
Os02g0182100 OsRR17
Os02g0796500 OsRR18
Os03g0224200 OsRR19
Os06g0183100 OsRR20
-3
0
3

Figure S6

A

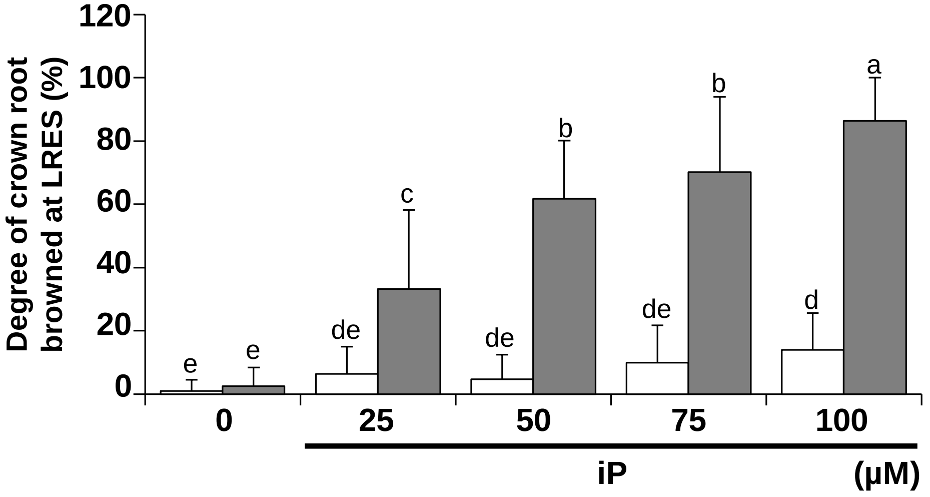
Degree of crown root browned at LRES (%)
120
100
80
60
40
20
0
e
e
de
c
de
b
de
b
d
a
0
25
50
75
100
iP
(μM)

B

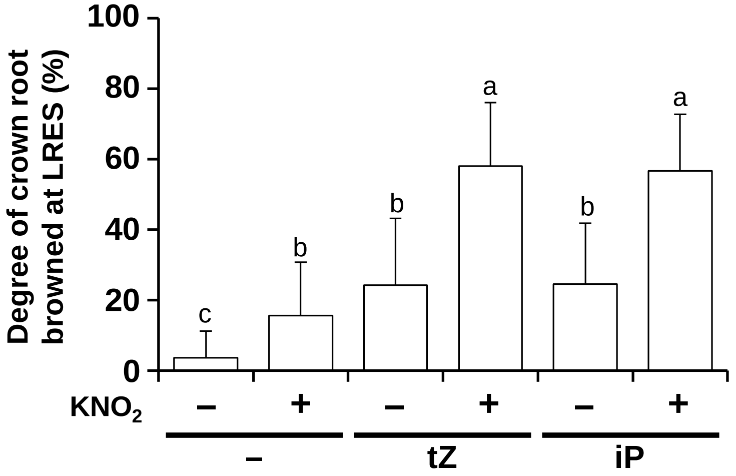
Degree of crown root browned at LRES (%)
100
80
60
40
20
0
c
b
b
a
b
a
KNO_2
–
+
–
+
–
+
–
tZ
iP

Figure S7

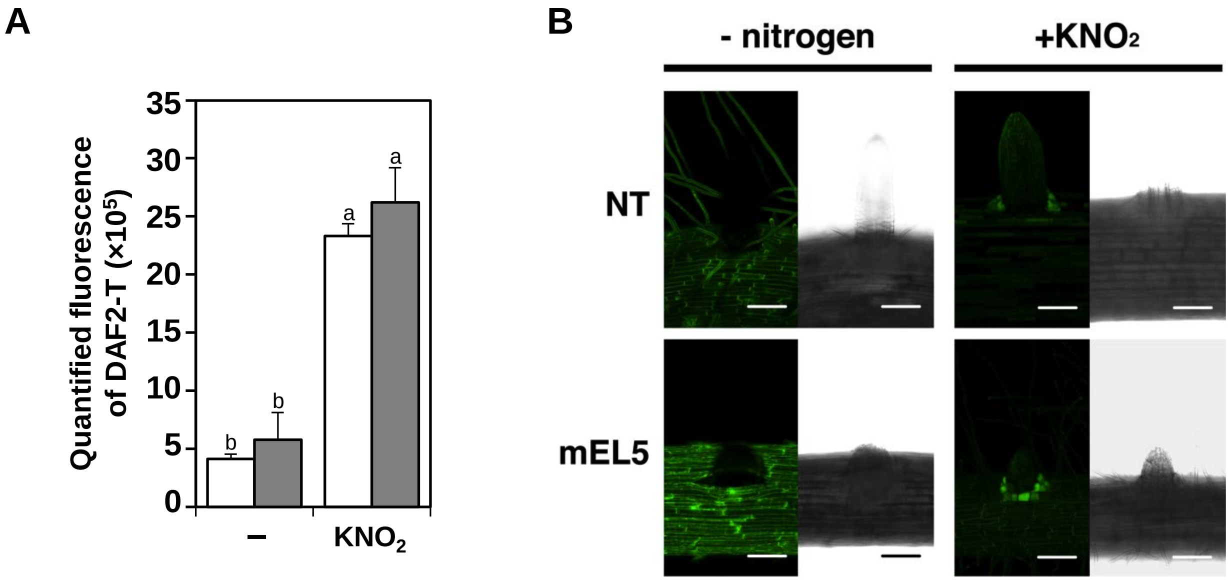

A
Quantified fluorescence of DAF2-T (×10^5)
35
30
25
20
15
10
5
0
b
b
a
a
–
KNO2
B
- nitrogen
+KNO2
NT
mEL5

Figure S8

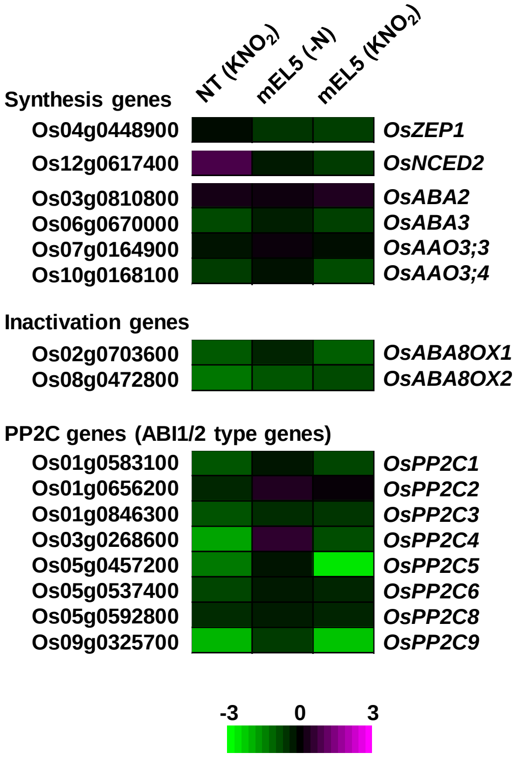

NT (KNO_2)
mEL5 (-N)
mEL5 (KNO_2)
Synthesis genes
Os04g0448900
Os12g0617400
Os03g0810800
Os06g0670000
Os07g0164900
Os10g0168100
OsZEP1
OsNCED2
OsABA2
OsABA3
OsAAO3;3
OsAAO3;4
Inactivation genes
Os02g0703600
Os08g0472800
OsABA8OX1
OsABA8OX2
PP2C genes (ABI1/2 type genes)
Os01g0583100
Os01g0656200
Os01g0846300
Os03g0268600
Os05g0457200
Os05g0537400
Os05g0592800
Os09g0325700
OsPP2C1
OsPP2C2
OsPP2C3
OsPP2C4
OsPP2C5
OsPP2C6
OsPP2C8
OsPP2C9
-3
0
3

Figure S9
